# Cucurbit[8]uril-based supramolecular theranostics

**DOI:** 10.1186/s12951-024-02349-z

**Published:** 2024-05-09

**Authors:** Dan Wu, Jianfeng Wang, Xianlong Du, Yibin Cao, Kunmin Ping, Dahai Liu

**Affiliations:** 1 Department of Vascular Surgery, China-Japan Union Hospital, Jilin University, Changchun, 130033 People’s Republic of China; 2 Department of Radiotherapy, China-Japan Union Hospital, Jilin University, Changchun, 130033 People’s Republic of China; 3 College of Materials Science and Engineering, Zhejiang University of Technology, Hangzhou, 310014 People’s Republic of China; 4 Bethune First Clinical Medical College, Jilin University, Changchun, 130012 People’s Republic of China

**Keywords:** Cucurbit[8]uril, Host–guest reactions, Self-assembly, Supramolecular nanomedicine, Supramolecular theranostics

## Abstract

Different from most of the conventional platforms with dissatisfactory theranostic capabilities, supramolecular nanotheranostic systems have unparalleled advantages via the artful combination of supramolecular chemistry and nanotechnology. Benefiting from the tunable stimuli-responsiveness and compatible hierarchical organization, host–guest interactions have developed into the most popular mainstay for constructing supramolecular nanoplatforms. Characterized by the strong and diverse complexation property, cucurbit[8]uril (CB[8]) shows great potential as important building blocks for supramolecular theranostic systems. In this review, we summarize the recent progress of CB[8]-based supramolecular theranostics regarding the design, manufacture and theranostic mechanism. Meanwhile, the current limitations and corresponding reasonable solutions as well as the potential future development are also discussed.

**Graphical Abstract:**

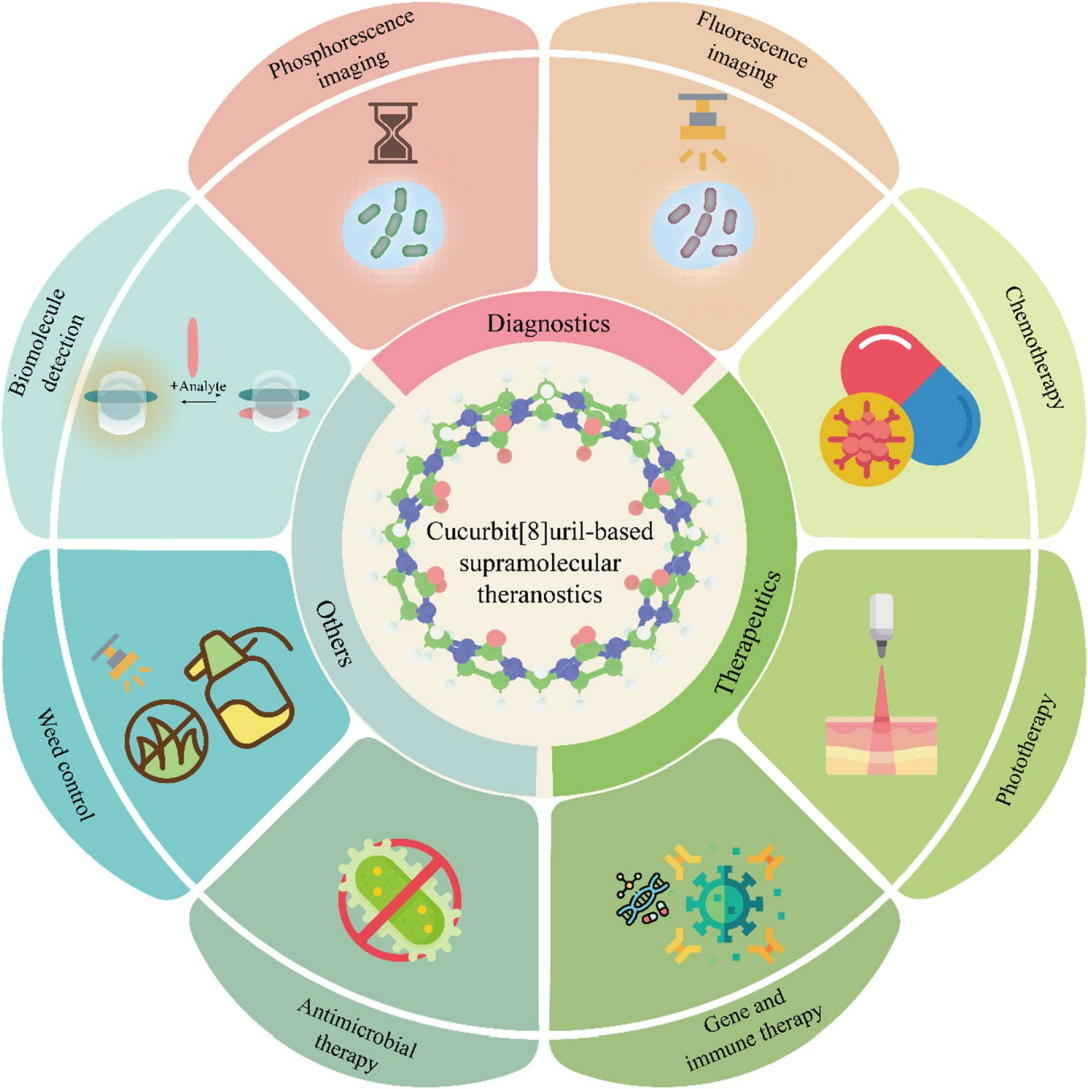

## Introduction

Improving the living quality is always the enthusiasm of human beings. However, all kinds of diseases persist in haunting people during their lives, bringing damages to bodies and sometimes even death [1]. Scientific community has committed to develop novel therapeutic medicines and approaches to boost drug effectiveness and reduce the grim side effects [2–6], aiming to harvest more in-depth understanding of life course and accordingly improve life quality [7–9].

Compared with the traditional nanotheranostic systems, supramolecular nanotheranostic systems exhibit unique properties attributing to their dynamic responsiveness of non-covalent interactions [10–26]. Various non-covalent interactions, such as Van der Waals force, hydrogen bonding, π–π stacking interactions, electrostatic interactions, metal–ligand coordinations and charge-transfer interactions, have been widely employed to construct supramolecular theranostic entities via hierarchically assembling building blocks. Especially, host–guest recognition reactions, another conspicuous non-covalent interaction, are drawing increasing attentions owing to their high binding affinities which can be regulated by external stimuli including pH, temperature, ion, enzyme, redox and light. Interestingly, some of these stimuli are the specificity of lesional microenvironment. Consequently, smart supramolecular theranostic systems can release the loaded cargoes specifically in the active sites, efficiently impairing the systemic toxicity of free drugs [27–31]. Additionally, host–guest inclusions also greatly enhance the solubility/stability of drugs [32–34] and avoid the tedious and time-consuming synthesis with the help of the “Lego-like” incorporation manner [35–39].

Cucurbit[*n*]urils (CB[*n*]s or Q[*n*]s, *n* = 5, 6, 7, 8, 10, 13, 14 and 15) are classical macrocyclic hosts prepared by the acid-catalyzed condensation of formaldehyde and glycoluril with a common depth (9.1 Å) and variational cavity sizes (2.4–11.0 Å) [40–45]. The hydrophobic cavity as well as two carbonyl-laced portals endow CB[*n*] with strong binding affinities towards a variety of guests (up to × 10^17^ M^–1^) [46], thus allowing the fabrication of stable CB[*n*]s-based supramolecular theranostic systems [47–58]. Nevertheless, supramolecular architectures based on CB[*n*]s (n = 5–7) are not easily constructed because these CB[*n*]s can only bind one guest. CB[8], with a large cavity, enables to simultaneously hold two homo/hetero guests in aqueous solution [59, 60], showing a great advantage on establishing supramolecular theranostic systems. Although CB[10] can also synchronously accommodate two guests, few works have been done so far [61–63], here we mainly discuss the CB[8]-based supramolecular theranostic organizations.

There have been a series of Reviews in the field of CB[*n*]-based supramolecular materials, such as supramolecular hydrogels [64], supramolecular switches [65], supramolecular polymers [66, 67], supramolecular frameworks [68], supramolecular amphiphiles [69], mechanically interlocked molecules [70] and supramolecular organic luminescent dyes [71], but the topic of CB[8]-based nanotheranostic systems has not been comprehensively summarized. This Review will fill this gap and systematically summarizes the progress in CB[8]-based theranostics (Table 1). By classifying the theranostic purposes, this Review is divided into three parts: supramolecular diagnose, supramolecular therapy and other applications including anti-bacteria, weeding and biomolecule detection. The sophisticated design of supramolecular theranostic systems and the diversiform therapeutic mechanisms will be discussed in detail. Furthermore, current limitations of supramolecular theranostic systems will be revealed, and reasonable solutions and potential future development will be proposed and prospected, respectively.

**Table 1:** Chemical structures of guest molecules referred here and their complex stoichiometry, treatment types and assembled structures

Chemical structures of guest	Complex stoichiometry	Therapy model	Composition structure
	*n:n*	A549	[78]
	*2:1*	A549	[90, 214]
	*1:1*	HeLa	[98]
	*1:1*	A549	[99]
	*1:2*	A549, HeLa, MCF-7, 293T	[106]
	*1:2*	A549, HeLa, KYSE-150 293T	[110]
	*1:1:1*	HeLa	[120]
	*1:1*	C32 B16F10 MCF-7 MDA-MB231	[132]
	*1:2*	GB A11T A13T A35T1	[139]
	*1:2*	HepG2	[126, 146]
	*1:2*	A549 S180	[152]
	*1:2*	SCC-7 COS7	[164]
	*1:2*	HepG2	[171]
	*1:2*	3T3 4T1 CT26	[175]
	*1:4*	B16F10	[181]
	*1:1*	*E. coli*	[187, 218]
	*1:1*	HEK 293 HepG2	[194]
	*1:3*	MCF-7	[200]
	*1:1:1*	DCs MCF-7	[206]
	*1:1:1*	HeLa HepG2	[233]
	*1:1:1*	Serum	[238]

## Cucurbit[8]uril-based supramolecular imaging

### Fluorescence imaging

**Fig. 1:**
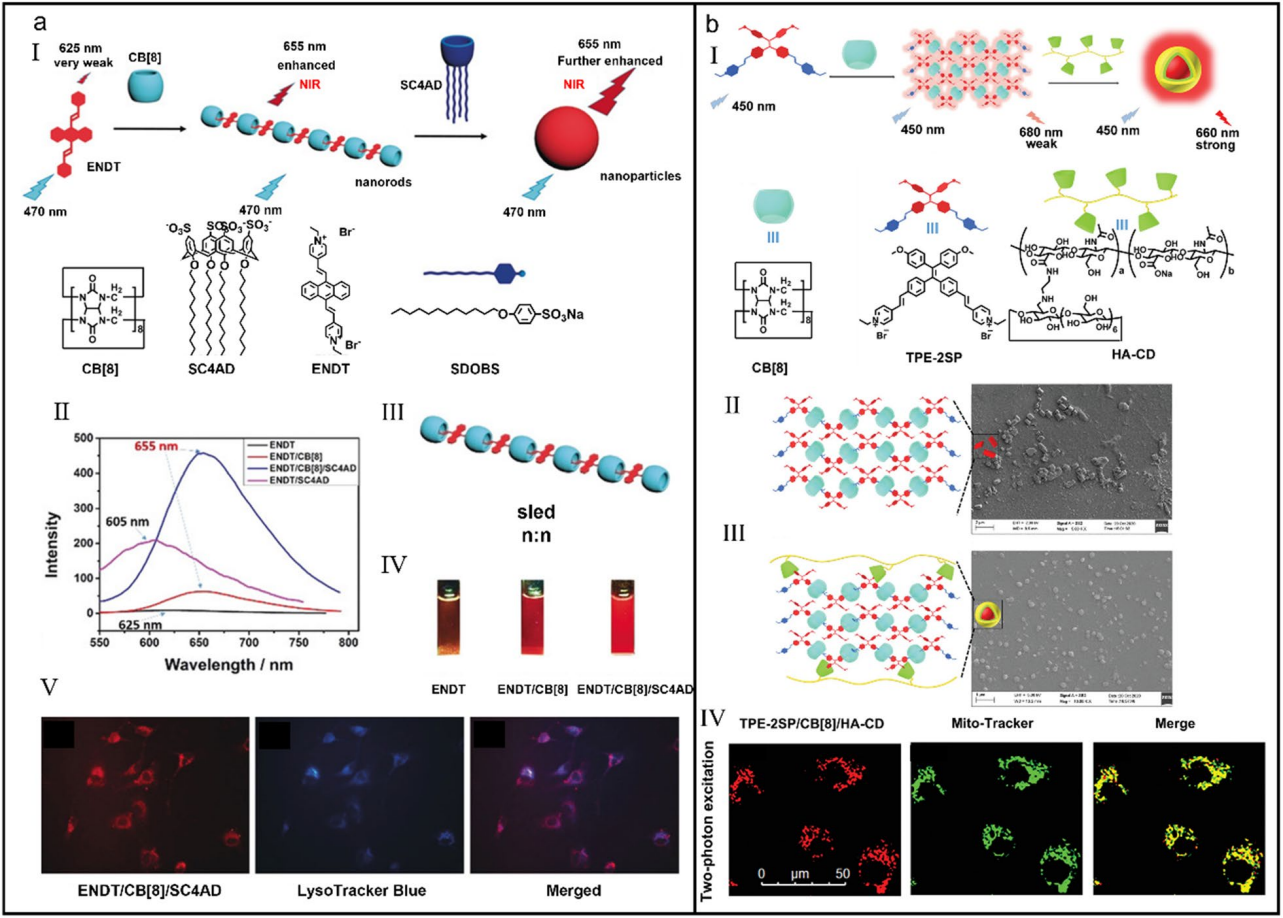
(**a**) Two-stage enhanced NIR supramolecular assemblies for cell imaging. (I) Illustration of the self-assemble process of NIR supramolecular assemblies. (II) Fluorescence emission spectroscopy of ENDT, ENDT/SC4AD, ENDT/CB[8] and ENDT/CB[8]/SC4AD. (III) Illustration of the sled *n:n* binding motif. (IV) Fluorescence photographs of ENDT, ENDT/CB[8] and ENDT/CB[8]/SC4AD. (V) Confocal laser scanning microscopy (CLSM) images of A549 cells treated with ENDT/CB[8]/SC4AD and LysoTracker Blue. Reproduced with permission [78]. Copyright 2018 Wiley–VCH Verlag GmbH & Co. KGaA, Weinheim (**b**) NIR supramolecular assemblies for two-photon targeting imaging. (I) Illustration of the formation of NIR supramolecular nanoparticles and cartoons of each component. Assembly schematic diagram and SEM images of TPE-2SP/CB[8] (II) and TPE-2SP/CB[8]/HA-CD (III). (IV) CLSM images of A549 cells treated with TPE-2SP/CB[8]/HA-CD and Mito-Tracker Green. Reproduced with permission [90]. Copyright 2021 Wiley–VCH GmbH

Benefiting from the advantages of slight photo-damage, deep tissue penetration and low background interference, near-infrared (NIR) fluorescent assemblies are widely applied in cell imaging [72–77]. Nevertheless, because the emission of most organic dyes cannot reach NIR region and the existence of aggregation induced quenching (ACQ) effect, NIR fluorescent assemblies are not easily available. Based on the host–guest complex ability of CB[8] and the calixarene-induced aggregation (CIA), Liu et al*.* developed a two-stage enhanced NIR supramolecular assembly for cell imaging (Fig. 1a-I) [78]. Attributing to the intermolecular charge transfer (ICT) between pyridinium and anthracene group and π–π stacking of anthracene groups, 4,4’-anthracene-9,10-diylbis(ethene-2,1-diyl)bis(1-ethylpyridin-1-ium) bromide (ENDT) emitted weak fluorescence at 625 nm (Fig. 1a-II). However, upon inclusion into CB[8] via a sled *n:n* binding motif (*K*_a_ = (1.04 ± 0.12) × 10^6^ L mol^_1^) (Fig. 1a-III), ENDT underwent *J*-aggregation and then ENDT/CB[8] assembled into nanorods, which initiated the first-stage emission enhancement and a red-shift (to 655 nm). When lower-rim dodecyl-modified sulfonatocalix[4]arene (SC4AD) was added into the solution of ENDT/CB[8], SC4AD assembled with nanorods to limit the intermolecular rotation of ENDT and provided a hydrophobic environment to further augment the emission of ENDT, both of which triggered the second-stage enhancement of fluorescence (Fig. 1a-IV). In vitro, ENDT/CB[8]/SC4AD not only showed negligible cytotoxicity but also lighted the lysosome of tumor cells (Fig. 1a-V), displaying a potential for lysosome-targeted cell imaging.

Despite great efforts have been made to enhance the emission of aggregation-induced emission fluorogens (AIEgens) with the aid of supramolecular macrocycle hosts [79–81], short absorption/emission wavelengths are still the puzzles greatly limiting their biomedical applications [82–84]. Two-photon excitation fluorophores which are usually located in the near-infrared region are equipped with the deep tissue penetration capacity, minimum background interference, high signal-to-noise ratio, and are preference for bioimaging [85–89]. Construction of supramolecular assemblies with two-photon excitation and NIR emission may provide more possibilities for medical imaging. Liu et al*.* reported a two-photon NIR supramolecular assembly based on tetraphenylethene derivative (TPE-2SP), CB[8] and β-cyclodextrin (β-CD)-modified hyaluronic acid (HA-CD) for mitochondria-targeted imaging (Fig. 1b-I) [90]. TPE-2SP emitted a weak fluorescence at 650 nm, but exhibited an enhanced fluorescence emission at 660 nm after complexing with CB[7]. Unlike the 1:2 supramolecular pseudorotaxane formed by TPE-2SP and CB[7], the self-assembly between TPE-2SP and CB[8] resulted in a two-axial netlike pseudopolyrotaxane which owned a close packing mode (*K*_a_ = 1.50 × 10^6^ M^_^^1^) (Fig. 1b-II) and triggered a redshift of 30 nm. Interestingly, TPE-2SP/CB[8] could further assemble into nanoparticles with the aid of HA-CD (Fig. 1b-III), which further boosted their NIR emission. Surprisingly, TPE-2SP/CB[8]/HA-CD-based supramolecular nanoparticles owned a two-photon character and were successfully engaged in mitochondrial targeting imaging (Fig. 1b-IV). This two-photon supramolecular system with assembly-induced stepwise enhancement of NIR luminescence opens a new way for targeted imaging.

### Phosphorescence imaging

Due to the large Stokes shift and long-lived photoemission, phosphorescence materials have attracted great interests in optical fields. Notably, phosphorescence materials in solution-phase are of particular interest for time-resolved biological imaging because their phosphorescence can be easily distinguished from the background fluorescence and auto-fluorescence in cellular biospecies [91–94]. Nevertheless, water and dissolved oxygen tend to induce the excited triplet state of phosphors to occur non-radiative relaxation decay [95–97], which leads to the phosphorescence quenching. Therefore, developing water-favoring phosphorescence systems is highly demanded, peculiarly with NIR emissive property.

**Fig. 2:**
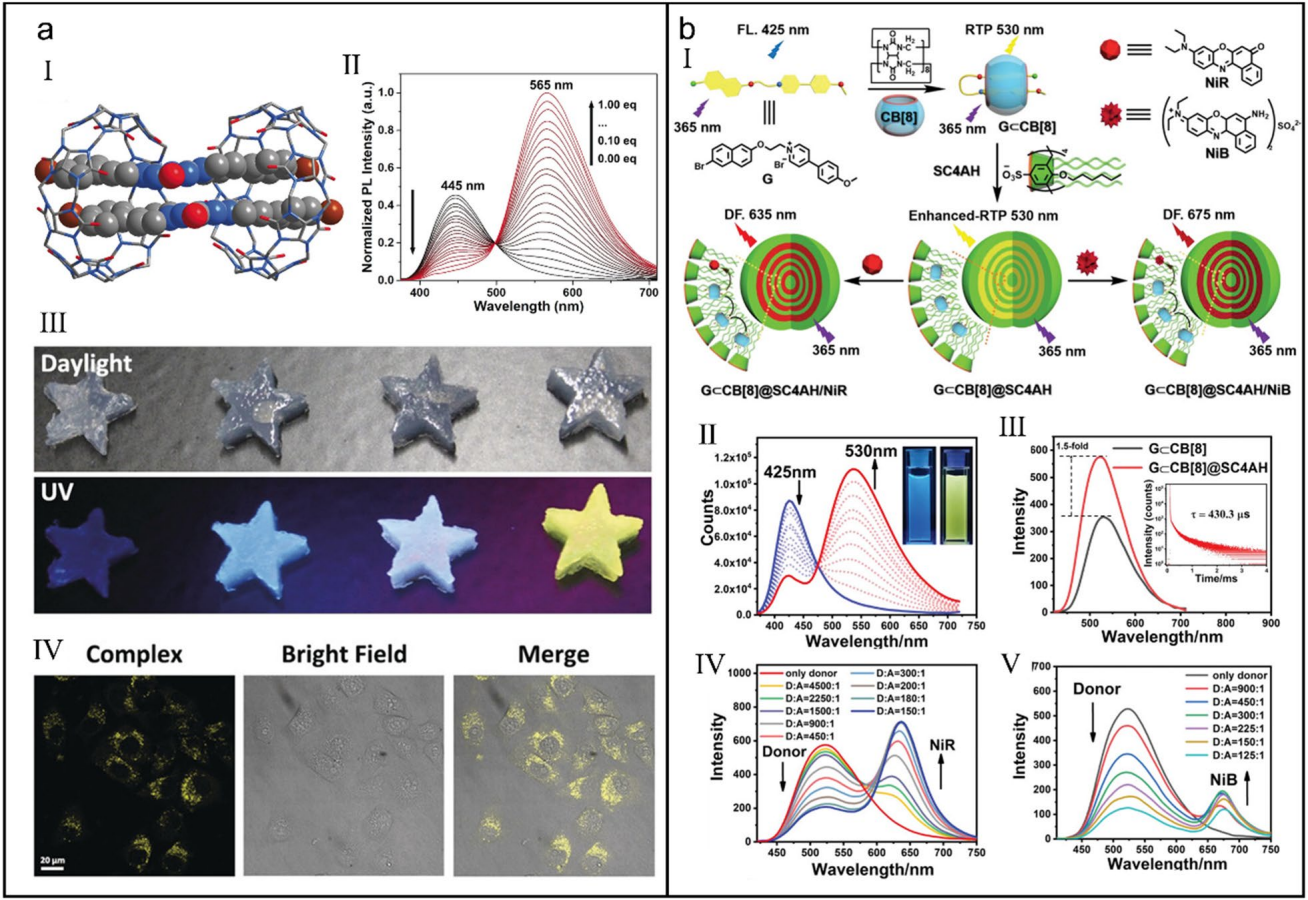
(**a**) Room-temperature phosphorescence emissive supramolecular assembly excited by visible-light. (I) X-ray diffraction single-crystal structure of supramolecular assembly (TBP)_2_·CB[8]_2_. (II) Phosphorescent emission spectra of TBP with gradual addition of CB[8]. (III) Photographs of hydrogels with different ratios of TBP and CB[8] under daylight or UV light. (IV) CLSM images of Hela cells incubated with TBP and·CB[8] (1:1). Reproduced with permission [98]. Copyright 2019 Wiley–VCH Verlag GmbH & Co. KGaA, Weinheim. (**b**) Supramolecular phosphorescence-capturing assembly for NIR lysosome imaging. (I) Illustration of the establishment of RTP-capturing system featured with a delayed NIR emission. (II) Phosphorescent emission spectra of G with gradual addition of CB[8]. (III) Phosphorescent emission spectra of G⊂CB[8]. Inset: The time-resolved phosphorescence decay plot of G⊂CB[8] at 530 nm. Phosphorescent emission spectra of G⊂CB[8]@SC4AH/NiR (IV) and G⊂CB[8]@SC4AH/NiB (V) at different ratios of donor and acceptor. Reproduced with permission [99]. Copyright 2021 Wiley–VCH GmbH

Tian et al*.* reported the first instance of visible-light-excited room-temperature phosphorescence (RTP) in aqueous phase using a host–guest assembly strategy [98]. The 2:2 quaternary model (Fig. 2a-I) of TBP-CB[8] complex induced a noteworthy redshift in the absorption (from 346 to 360 nm) and phosphorescence emission (from 445 to 565 nm) (*K*_a_ = 1.54 × 10^6^ M^_1^) (Fig. 2a-II). The mechanism was proposed that hydrogen bonding, diode–diode interaction and hydrophobic interaction triggered the CB[8]-directed stacking patterns, which not only efficiently restrained the molecular motion of TBP but also stably promoted the charge-transfer process with a redshifted visible-light wavelength. This unique CB[8]-mediated quaternary stacking mode allowed the visible-light excitation and tunable photoluminescence, enabling the engineered machining of multicolor hydrogels (Fig. 2a-III) and biological cell imaging (Fig. 2a-IV).

Based on two kinds of macrocyclic molecules, CB[8] and amphiphilic calixarene p-sulfonatocalix[4]arene tetrahexyl ether (SC4AH), Liu et al*.* constructed a phosphorescence capturing system with a delayed NIR emission via the secondary assembly strategy (Fig. 2b-I) [99]. Because CB[8] offered an independent cavity to enhance the intramolecular charge transfer (ICT) between methoxyphenyl pyridinium salt and naphthalene (*K*_a_ = 1.26 × 10^7^ M^_1^), intersystem cross (ISC) was improved and long-lived triplet state was obtained, which triggered a delayed phosphorescence emission at 530 nm (Fig. 2b-II). Moreover, owing to the further restraint of non-radiative relaxation via the secondary assembly with SC4AH, the phosphorescence emission of G⊂CB[8]@SC4AH was further enhanced (Fig. 2b-III). Interestingly, two phosphorescence-capturing systems with NIR emission at 635 (Fig. 2b-IV) and 675 nm (Fig. 2b-V), respectively, were feasibly acquired by introduction of Nile Red (NiR) or Nile Blue (NiB) as acceptor. More importantly, G⊂CB[8]@SC4AH/NiB not only held low cytotoxicity but also realized lysosome-targeted NIR imaging of tumor cells, providing a new multistage assembly approach for NIR imaging of living cells.

Liu et al*.* also reported other similar supramolecular assemblies emitting room-temperature phosphorescence on the basis of host–guest interaction and the secondary assembly strategy [100]. Benefiting from the energy transfer between supramolecular assembly and fluorescent dyes, delayed fluorescence was further observed in supramolecular assembly system, which was successfully applied in cell imaging.

**Fig. 3:**
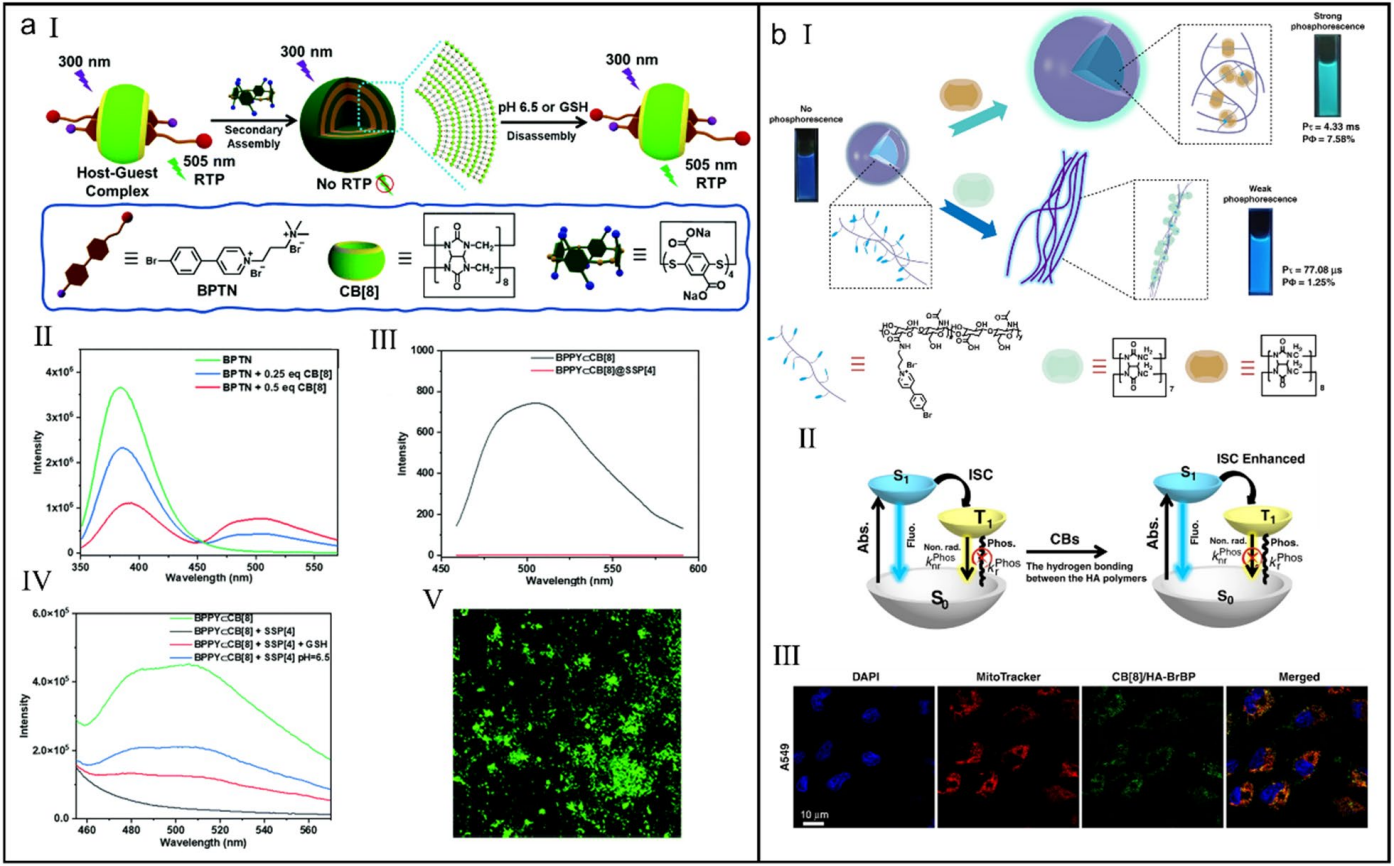
(**a**) Phosphorescent biaxial pseudorotaxane for selectively imaging tumor cells. (I) Illustration of the supramolecular assembly and disassembly of biaxial pseudorotaxane. (II) Photoluminescence spectra of BPTN in the presence of different concentrations of CB[8]. (III) Photoluminescence spectra of BPTNC⊂CB[8] and BPTNCCB[8]⊂SSP[4]. (IV) Photoluminescence spectra of BPTNC⊂CB[8], BPTNCCB[8]⊂SSP[4], BPTNCCB[8]⊂SSP[4] + GSH and BPTNCCB[8]⊂SSP[4] + weak acid. Reproduced with permission [106]. Copyright 2022 The Author(s) Published by the Royal Society of Chemistry. (**b**) Ultralong phosphorescence supramolecular polymer for tumor cell imaging. (I) Illustration of the construction of CBs/HA-BrBP supramolecular polymers. (II) The proposed mechanism of ultralong phosphorescence of supramolecular polymer. (III) CLSM images of A549 cells treated with CB[8]/HA-BrBP. Reproduced with permission [110]. Copyright 2020 The Author(s)

Considering the special profiles of pathological tissue microenvironment, those diagnostic reagents responsive to the lesional microenvironment can obtain more precise theranostic results [101–105]. Liu et al*.* constructed a biaxial pseudorotaxane supramolecular phosphorescent probe with pH and glutathione (GSH)-responsiveness on the basis of the CB[8]-mediated host–guest interaction and the secondary assembly with disulfide-pillar[4]arene (SSP[4]) (Fig. 3a-I) [106]. Upon formation of biaxial pseudorotaxane following a 1:2 complexing mode between CB[8] and SSP[4] (*K*_a_ = (2.02 ± 0.52) × 10^6^ M^_^1), the fluorescence of BPTN gradually disappeared and a new phosphorescence emission at around 505 nm emerged (Fig. 3a-II). Further assembly with SSP[4] led to the quenching of phosphorescence (Fig. 3a-III). However, low pH and GSH could induce the disassembly of non-phosphorescent assembly and in turn recover its phosphorescence (Fig. 3a-IV), which was nicely used for specifically imaging tumor cells which are featured with low pH and high concentration of GSH (Fig. 3a-V).

Although host–guest interactions have promoted the development of phosphorescence in water phase, the phosphorescence systems with a long lifetime are rarely reported [107–109]. Liu et al*.* constructed two water-soluble phosphorescence supramolecular polymers based on CB[7]/CB[8] and 4-(4-bromophenyl)pyridin-1-ium bromide (BrBP)-modified hyaluronic acid (HA) (HA–BrBP), in which pseudorotaxane polymer CB[7]/HA-BrBP self-assembled into nanofibers (*K*_a_ = (3.81 ± 0.22) × 10^6^ M^_1^) but biaxial pseudorotaxane polymer CB[8]/HA-BrBP existed as the large spherical aggregates (*K*_a1_ = (4.49 ± 0.19) × 10^5^ M^_1^ and *K*_a2_ = (2.43 ± 0.08) × 10^6^ M^_1^) (Fig. 3b-I) [110]. Benefiting from the synergistic effects of host–guest interaction and multiple hydrogen bonds, the molecular motion of CB[8]/HA-BrBP in aqueous solution was restricted, thus the ISC was promoted and meanwhile the non-radiative decay was reduced, which eventually contributed to a long phosphorescence lifetime up to 4.33 ms (Fig. 3b-II). Profiting by the targeting ability of HA, CB[8]/HA-BrBP was also successfully applied for the targeted phosphorescence imaging of cancer cells (Fig. 3b-III).

### Cucurbit[8]uril-based supramolecular therapeutics

#### Chemotherapy

Chemotherapy, as the most common performed procedures to treat a variety of diseases, faces a variety of challenges in clinical applications, such as the poor specificity, low bioavailability and severe side effects [111–115]. Nanoparticles constructed from polymeric matrix have become brilliant drug delivery systems (DDSs) owing to their excellent biodegradability and biocompatibility [116–119]. However, the mission of traditional DDSs is only to transport therapeutic drugs, it is necessary to develop precise treatments such as stimuli-responsive drug release and imaging-guided therapy.

**Fig. 4:**
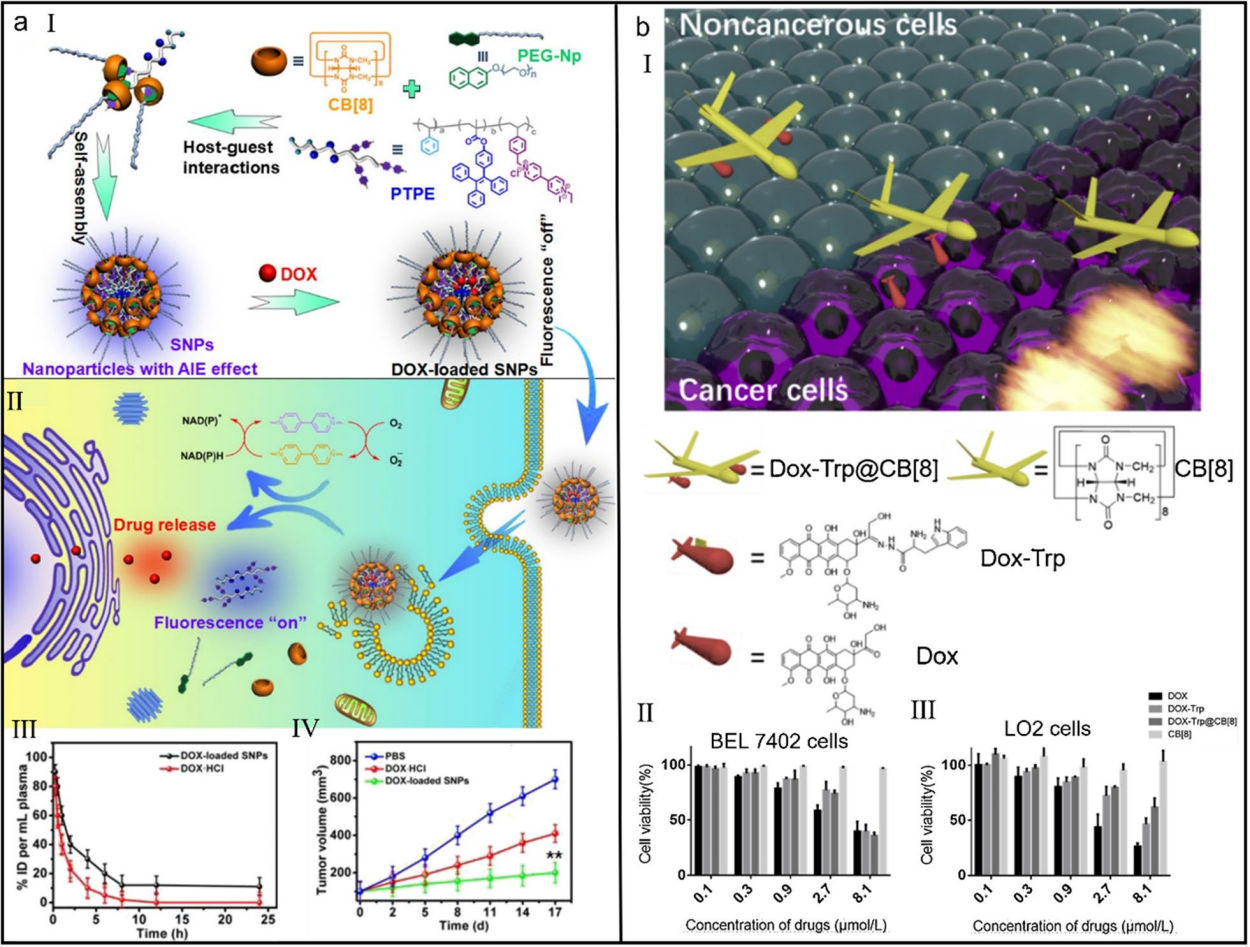
(**a**) CB[8]-based supramolecular nanomedicine for tumor therapy. (I) Chemical structures of different building blocks and the preparation of supramolecular nanomedicine. (II) Illustration of the imaging-guided selective drug release. (III) Pharmacokinetics of free DOX and DOX-loaded SNPs. (IV) Tumor volume change of mice with different treatments. Reproduced with permission [120]. Copyright 2017 American Chemical Society. (**b**) Supramolecular DOX-dimer for selective drug release. (I) Chemical structures of different building blocks and the construction of supramolecular dimeric prodrug. Cell viability of BEL 7402 cells (II) and LO2 cells (III) after different treatments. Reproduced with permission [126]. Copyright 2019 Chinese Chemical Society and Institute of Materia Medica, Chinese Academy of Medical Sciences. Published by Elsevier B.V. All rights reserved

Tang et al*.* constructed a supramolecular nanomedicine for imaging-guided cancer therapy [120]. Based on the host−guest molecular recognition reaction between CB[8], 4,4′-bipyridinium derivative (PTPE) and PEGylated naphthol (PEG-Np), amphiphilic supramolecular brush copolymer CB[8] ⊃ (PEG-Np·PTPE) was established, which self-assembled into supramolecular nanoparticles in aqueous solution. Hydrophobic chemotherapeutic drug DOX was sealed in the hydrophobic core of supramolecular nanoparticles, establishing a supramolecular nanomedicine with Förster resonance energy transfer effect (Fig. 4a-I). Under the stimulation of low pH and intracellular reducing agents, supramolecular nanomedicine realized the controlled drug release in tumor microenvironment (Fig. 4a-II). Benefiting by the supramolecular self-assembly, supramolecular nanomedicine was highly accumulated in tumor tissues via the EPR effect and possessed a long half-life period (Fig. 4a-III), which contributed to a satisfying antitumous effect (Fig. 4a-IV).

Owing to excellent guest-binding behaviors, CB[8] has been frequently engaged as a non-covalent crosslinking agent in the construction of various functional materials [121–125]. However, in almost all of these cases, CB[8] was used as crosslink for polymers, peptides and proteins, whereas CB[8]-cross-linked dimeric prodrug is extremely rare. Based on the 1:2 host–guest complexation between CB[8] and Tryptophan (Trp) (*K*_a1_ = 1.99 × 10^6^ M^_1^ and *K*_a2_ = 1.14 × 10^5^ M^_1^), Wang et al*.* designed a supramolecular dimeric prodrug, in which DOX was linked to Trp via an acid-labile hydrazone bond, realizing the pH-responsive DOX release in tumor cells (Fig. 4b-I) [126]. Under this strategy, other drugs can be chemically modified and carried as double guns into “warplane”-like CB[8] for improving cancer therapy (Fig. 4b-II and III).

**Fig. 5:**
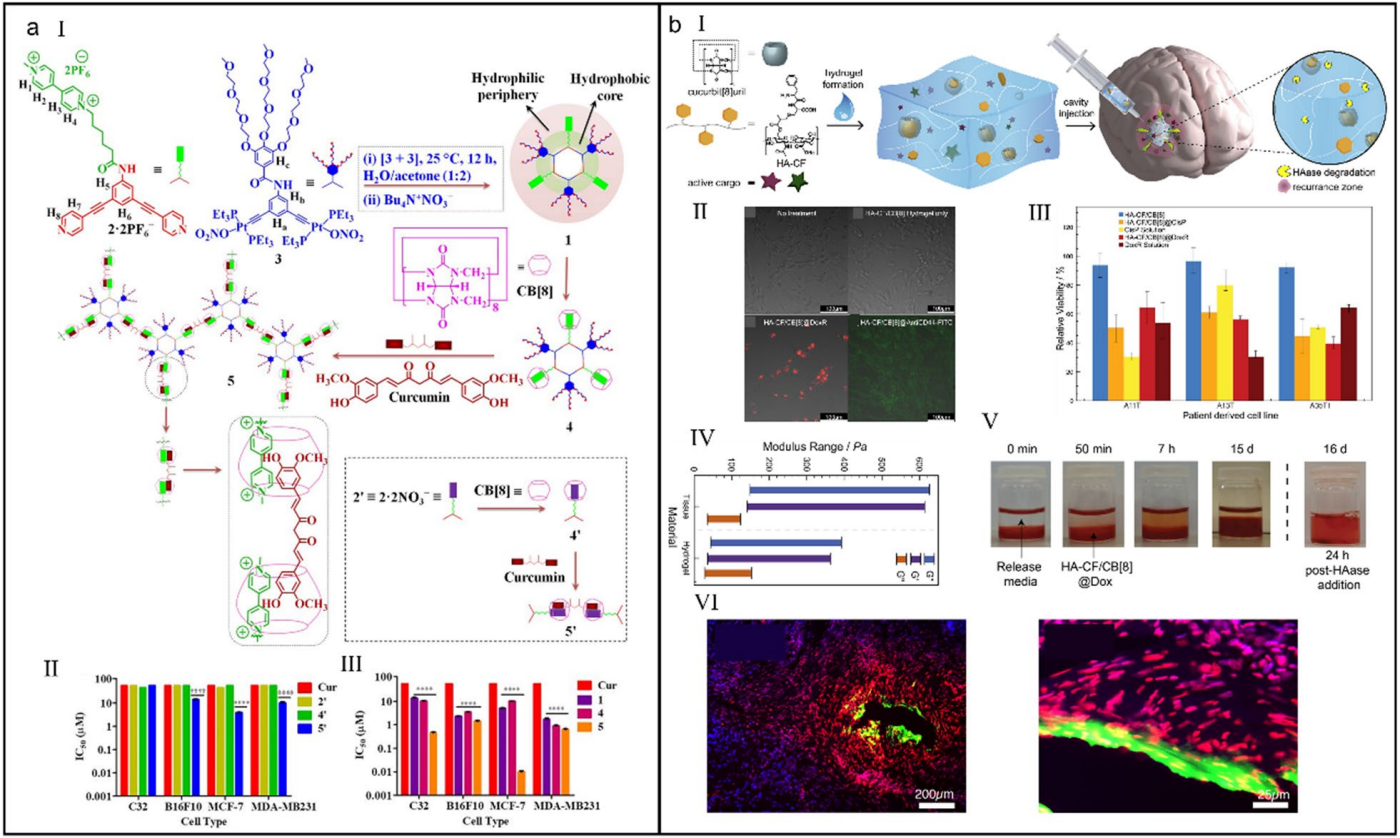
(**a**) Orthogonal organoplatinum(II) metallacycle for tumor therapy. (I) Schematic illustration of the self-assembly of supramolecular system. (II) IC_50_ value of 2', 4' and 5' measured on different cell lines. (III) IC_50_ value of 1, 4 and 5 measured on different cell lines. Reproduced with permission [132]. Copyright 2018 Published under the PNAS license. (**b**) A CB[8]-based hydrogel delivery vehicle for GB therapy. (I) Illustration of the preparation of supramolecular hydrogel and its cure mechanism. (II) Fluorescence images of GB cells after different treatments. (III) Cell viability of different cells after different treatments. (IV) The moduli comparation between tissue and supramolecular hydrogel. (V) The stability study of supramolecular hydrogel. (VI) The immumohistochemical staining of GB tissue reflecting the tissue penetrability of supramolecular hydrogel delivery vehicle. Reproduced with permission [139]. Copyright 2018 Published by Elsevier Ltd

Despite the recent breakthrough in cancer research, the improvement of the solubility of hydrophobic drugs in water is still a stubborn challenge. The orthogonality of different noncovalent interactions has been proved to be a facile method to improve the water solubility of drug and realize controlled drug release [127–130]. Nevertheless, most of the known supramolecular orthogonal system are prepared in organic medium, severely limiting their relevant biomedical applications [131]. Stang et al*.* combined bis-phosphine organoplatinum(II) ← pyridyl metal–ligand coordination and CB[8]/MV-directed host–guest complexation (*K*_a1_ × *K*_a2_ = 10^9^–10^10^ M^_2^) to establish a water-soluble supramolecular system (Fig. 5a-I) which not only was able to complex with hydrophobic curcumin via a 1:1:1 complexing manner but also display a superior anticancer effect over free curcumin (Fig. 5a-II and III) [132].

Glioblastoma (GB) is one of the most aggressive malignant brain tumor in adults with a just 4.6 months of median survival [133–135]. Owing to the need of crossing blood–brain barrier (BBB) and sophisticated therapeutic environment, few chemotherapeutic agents meet the clinical treatment request of GB [136–138]. Scherman et al*.* developed a HA-CF/CB[8] hydrogel carrier specially for GB treatment (Fig. 5b-I) [139]. Attributing to the matched biocompatibility with surrounding tissue environment (Fig. 5b-II and III), continuous shape and structural remodeling were achieved, which was good for tissue healing. (Fig. 5b-IV). Furthermore, efficient degradation of gel (Fig. 5b-V) and deep penetrativity (Fig. 5b-VI) of the cargos into ex vivo tissue slice indicated the bright prospect of supramolecular hydrogel for future GB therapy.

**Fig. 6:**
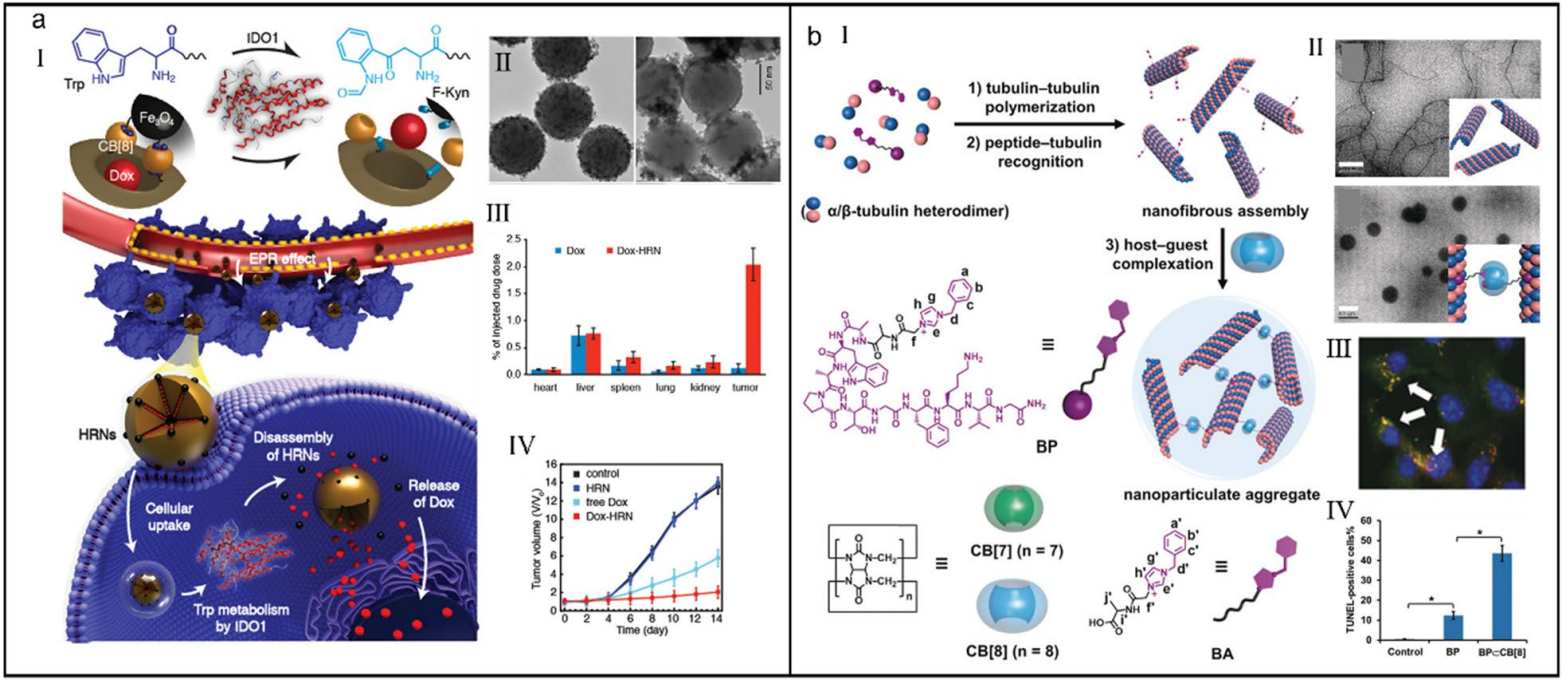
(**a**) Trp/CB[8]-mediated hybrid nanoparticles for targeted drug delivery in IDO1-overexpressed tumor cells. (I) Illustration of the targeted release mechanism of hybrid supramolecular nanoparticles. (II) Transmission electron microscope (TEM) images of hybrid nanoparticles (left) and their collapse upon exposure to IDO1 (right). (III) Biodistribution of DOX in major organs and tumors at 24 h post-injection of free DOX and hybrid supramolecular nanoparticles. (IV) Tumor volume change of mice during treatment. Reproduced with permission [146]. Copyright 2019 WILEY–VCH Verlag GmbH & Co. KGaA, Weinheim. (**b**) CB[8]-mediated microtubule aggregation for enhancing cell apoptosis. (I) Illustration of BP⊂CB[8]-mediated targeted microtubular aggregation. (II) TEM images of free MTs (up) and BP@MTs (down). (III) CLSM image of A549 cells treated with BP⊂CB[8]. (IV) The percentage of TUNEL-positive cells in tumor tissue of mice after different treatments. Reproduced with permission [152]. Copyright 2019 Wiley–VCH Verlag GmbH & Co. KGaA, Weinheim

Various enzymes are active and highly expressed in the tumor microenvironment, which can be utilized as tumor-specific stimuli to enhance the selectivity and sensitivity of drug delivery system [140–145]. However, the majority of the enzyme triggers are either expressed extracellularly or within organs, resulting in the random drug release and severe side effects. Hu et al*.* presented an enzyme-responsive hybrid drug delivery system, which released payload therapeutics solely in the presence of intracellular indoleamine 2,3-dioxygenase 1 (IDO1), diminishing premature drug release [146]. Trp was conjugated onto the surface of Fe_3_O_4_ nanoparticles and the hatchway of silica core, and drug-loaded raspberry-like nanoparticles were prepared based on the host–guest recognition between CB[8] and Trp (Fig. 6a-I). In the presence of IDO1, Trp was oxidized into *N*-formylkynurenine (F-Kyn), leading to the opening of channel gates of nanoparticles (Fig. 6a-II) and triggering the drug release specifically in tumor cells (Fig. 6a-III). Because of the high selectivity of nanocarrier to IDO1-overexpressed tumor cells, significant in vitro cytotoxicity and superior antitumor effects (Fig. 6a-IV) were acquired, providing a promising platform for accurate intracellular drug release.

Supramolecular methodology on the basis of cavity-bearing macrocycles has been proven as a powerful strategy to regulate the functions of many natural biomacromolecules [147, 148]. Microtubule (MT), a key protein filament of the cytoskeleton, plays critical roles in intracellular transport and cell division, gradually developing into absorbing molecular targets for biomolecular assemblies and cancer chemotherapy [149–151]. Liu et al*.* presented a supramolecular microtubular system by combing primary tubulin–tubulin heterodimerization, specific peptide–tubulin recognition and cooperative host–guest complexation to seek the curative effect of intertubular aggregation (Fig. 6b-I) [152]. An benzylimidazolium-bearing antimitotic polypeptide (BP) with tubulin-targeting ability provided a anchoring point to complex with CB[8], exclusively inducing the dramatic morphological changes of MT from linear polymers to spherical nanoparticles (*K*_a_ = (8.66 ± 0.43) × 10^5^ M^_1^) (Fig. 6b-II). After incubation with BP⊂CB[8], evident compact MTs were found in cellular environment (Fig. 6b-III) and a high level of apoptosis was induced in the tumor tissues (Fig. 6b-IV), demonstrating that orthogonal supramolecular interaction-enhanced intertubular aggregation provides a novel strategy for the fight against MT-related diseases.

#### Phototherapy

Compared with chemotherapy, photodynamic therapy (PDT) exhibits the non-invasiveness and high spatiotemporal controllability [153–156]. Photosensitizers (PSs) are the important component of PDT, which can use luminous energy to generate toxic reactive oxygen species (ROS) and then massively damage cells [157, 158].

**Fig. 7:**
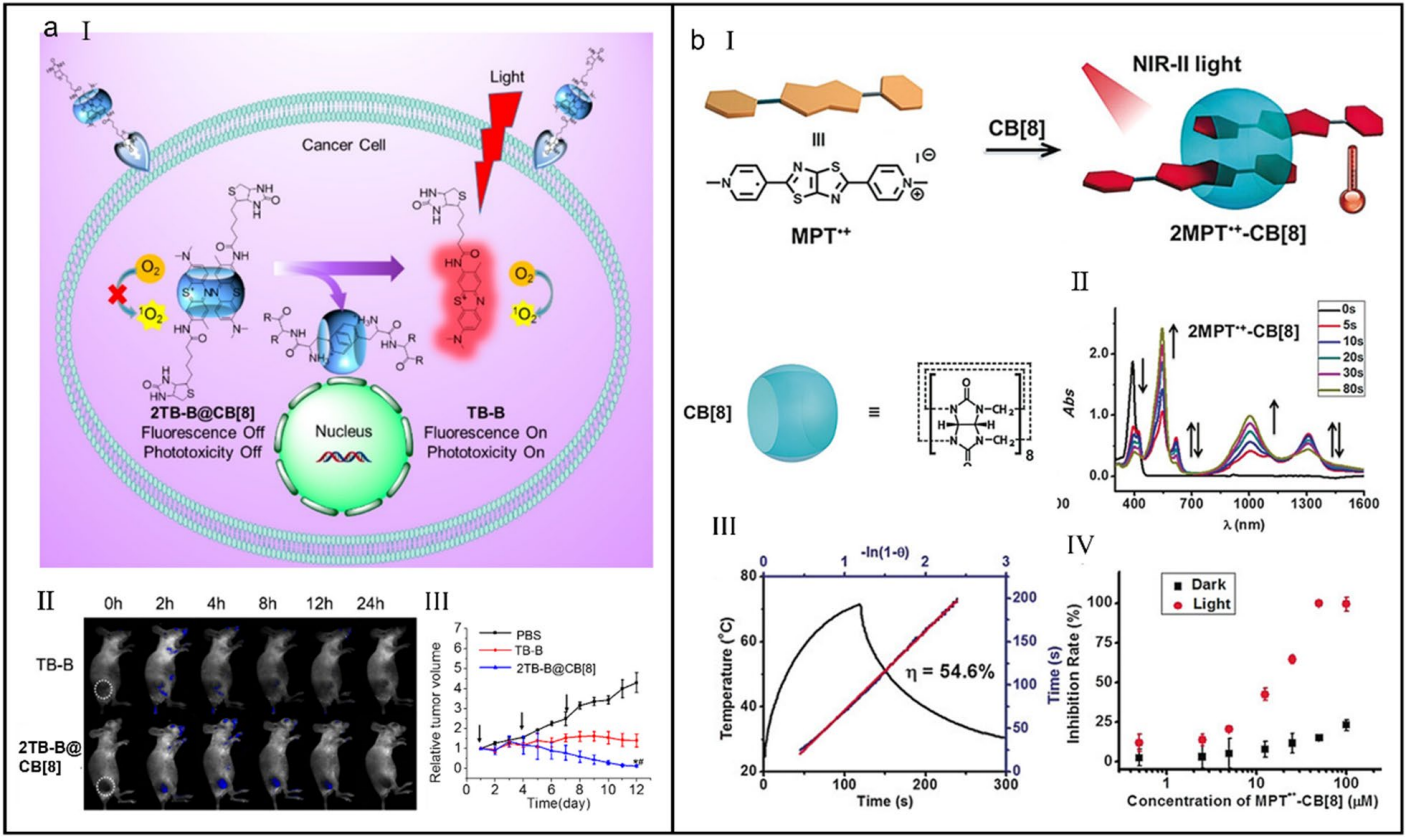
(**a**) CB[8]-regulated aPS for imaging-guided PDT. (I) Illustration of the mechanism of aPS-mediated imaging-guided PDT. (II) In vivo imaging of mice intravenously administrated with TB-B and 2TB-B@CB[8]. (III) Tumor volume change of mice during treatments. Reproduced with permission [164]. Copyright 2016 American Chemical Society. (**b**) A CB[8]-based supramolecular radical dimer with a high NIR-II photothermal conversion efficiency. (I) Illustration of the self-assembly of 2MPT^•+^-CB[8]. (II) UV/Vis–NIR spectra of 2MPT^•+^-CB[8] with different irradiation time. (III) Heating and cooling cycle of 2MPT^•+^-CB[8] and the calculated photothermal conversion efficiency. (IV) Inhibition rate plots of HepG2 cells after 2MPT^•+^-CB[8] induced PTT. Reproduced with permission [171]. Copyright 2019 Wiley–VCH Verlag GmbH & Co. KGaA, Weinheim

Current PSs can only indistinguishably carry on cell imaging and killing, not intelligent enough to fill the requirement of personalized treatment. Activatable photosensitizers (aPSs) which are activated by disease-related triggers hold great possibility for personalized PDT [159–163]. The most commonly used strategies for construction of aPSs are covalent modifications, which suffer from problems involving tedious synthesis and advance or lag of activation. Zhang et al*.* reported a CB[8]-regulated aPS for imaging-guided PDT (Fig. 7a-I) [164]. CB[8] can bind with biotinylated toluidine blue (TB-B) through host–guest interaction (*K*_a_ = 2.67 × 10^7^ M^_1^), and the fluorescence and PDT activity of TB-B can be turned on or off via the assembly/disassembly of 2TB-B@CB[8]. With the protection of CB[8], TB-B cannot be easily reduced by enzymes, thus enhancing the stability of TB-B in vivo (Fig. 7a-II) and eventually contributing to an improved anticancer behavior (Fig. 7a-III).

Benefiting from the maximum permissible exposure and excellent penetration depth, NIR photosensitizers have attracted growing interest in phototherapy [165–167]. Compared with NIR-I (750–1000 nm) photosensitizers, NIR-II (1000–1350 nm) photosensitizers exhibit better photo-conversion efficiency and biological tissue penetration [168–170]. However, the limited solubility, strong aggregation and fussy synthetic procedure limit their further biological applications. Zhang et al*.* developed a supramolecular strategy to realize a high-efficiency NIR-II PTT via fabricating supramolecular radical dimer [171]. Attributing to the acceptor–donor–acceptor configuration, *N*,*N’*-dimethylated dipyridinium thiazolo[5,4-d]thiazole (MPT^2+^) tended to form a supramolecular dimer inside the cavity of CB[8], forming a 2:1 host–guest inclusion (2MPT^2+^-CB[8]) (*K*_a1_ = 5.69 × 10^6^ M^_1^ and *K*_a2_ = 1.36 × 10^6^ M^_1^) (Fig. 7b-I). Upon MPT^2+^ was reduced into MPT^•+^, a strong NIR-II absorption could be achieved with the help of the CB[8]-enhanced ICT (Fig. 7b-II), which prompted a high-efficiency photothermal conversion (Fig. 7b-III). In addition, the stability of radical dimer was also improved with the shelter of CB[8], collectively contributing to a high inhibition rate of cancer cells (Fig. 7b-IV). This line of supramolecular research provides a new path to fertilize the application of organic radicals in phototherapy.

**Fig. 8:**
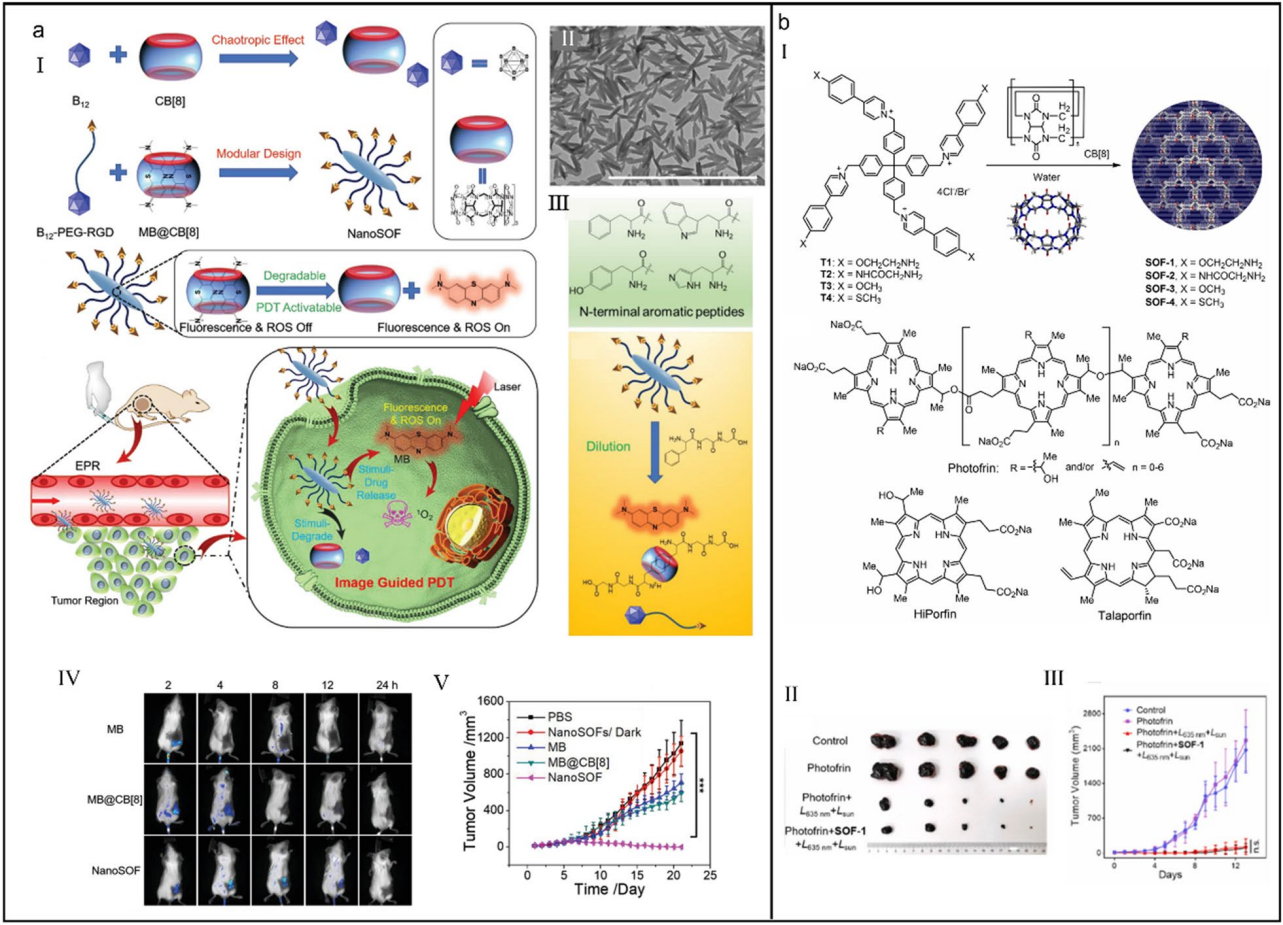
(**a**) CB[8]-regulated supramolecular organic frameworks for imaging-guided PDT. (I) Construction of CB[8]-regulated supramolecular organic frameworks and their application for imaging-guided PDT. (II) TEM image of the supramolecular organic framework. (III) The chemical structures of *N*-terminal aromatic peptides (up) and the illustration of dilution effect and *N*-terminal aromatic peptides-co-triggered degradation of supramolecular organic frameworks (down). (IV) In vivo fluorescence images of mice with different treatments. (V) Tumor volume change of mice with different treatments. Reproduced with permission [175]. Copyright 2020 Wiley–VCH GmbH. (**b**) Supramolecular organic frameworks applied to improve the safety of clinical porphyrin photosensitizers without breaking their antitumor efficacy. (I) Illustration of the formation of supramolecular organic frameworks. (II) Photos of excised tumor tissues of mice with different treatments. (III) Tumor volume change of mice with different treatments.. Reproduced with permission [181]. Copyright 2022 Elsevier Ltd. All rights reserved

Host–guest interaction-based supramolecular architectures have provided miscellaneous therapeutic schedules for diseases treatment, but the role of host is only to complex guest molecules or pharmaceutical molecules. It seems that the functionality of host molecules is millennially unchanged [172–174]. Wang et al*.* explored the chaotropic effect between closo-dodecaborate cluster (B_12_) and CB[8] to regulate the self-assembly of supramolecular organic frameworks (SOFs) and realize the targeted imaging and PDT (Fig. 8a-I) [175]. Chaotropic anions B_12_ are prone to interact with the positive polar and hydrophobic surfaces of CB[8], thus CB[8] could be further used to encapsulate methylene blue (MB) via host–guest interaction (*K*_a_ = 3.24 × 10^13^–2.50 × 10^16^ M^_2^) for PDT. When B_12_-PEG-RGD met with MB@CB[8] in water, a shuttle-shaped NanoSOF was formed (Fig. 8a-II), which could accumulate in tumor tissue with the synergistic effect of targeting peptide RGD and the enhanced permeability and retention (EPR). When entering into tumor cells, intracellular substances carrying *N*-terminal aromatic peptides triggered the release of MB from NanoSOF (Fig. 8a-III) and the imaging-guided PDT was realized (Fig. 8a-IV and V). This work emphasizes the architectonic regulatory function of chaotropic effect, extending the inclusion property of CB[8] and providing more possibilities for construction of various supramolecular self-assemblies used in other fields.

Although PDT has developed into the major treatment modality for skin diseases and cancer [176–178], the skin photosensitivity caused by the body accumulation of clinical photosensitizers is still the unsolved number-one priority, which brings much trouble and poor quality of life for patient [179, 180]. Li et al*.* reported a three-dimensional supramolecular organic frameworks to reduce the skin phototoxicity of three clinical porphyrin-based photodynamic agents (PDAs) based on an adsorption and retention mechanism (Fig. 8b-I) [181]. Skin lesion experiments demonstrated that supramolecular organic frameworks remarkably suppressed the sunlight-tempted skin phototoxicity and tumor-bearing mouse model proved that the efficacy of PDT posted by supramolecular organic frameworks was still high (Fig. 8b-II and III), collectively certifying that this supramolecular organic frameworks provided an efficient strategy to improve the safety of clinically applied PDAs.

#### Gene and immune therapy

**Fig. 9:**
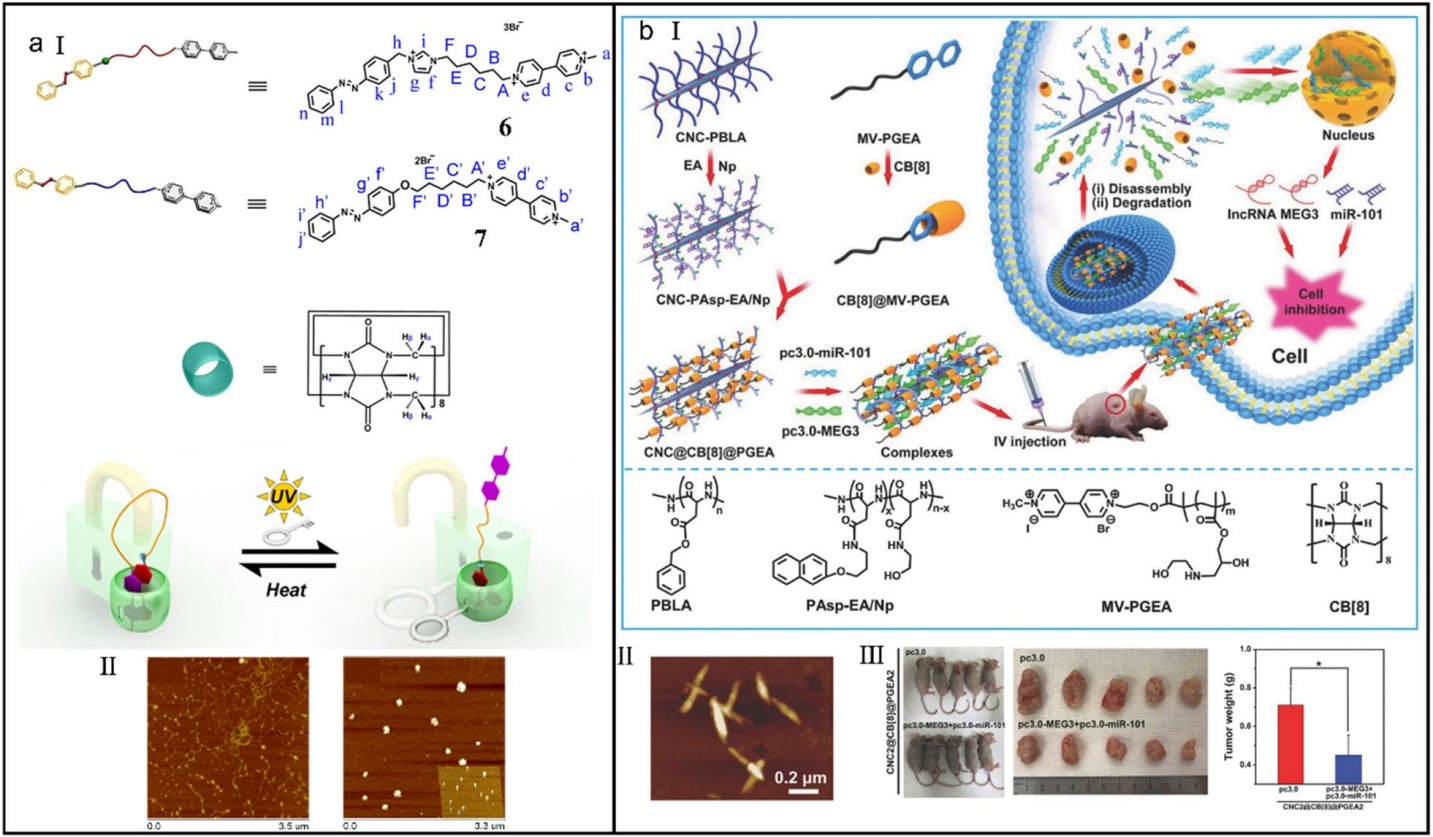
(**a**) Photoresponsive supramolecular complexes for efficiently regulating DNA. (I) Chemical structures of 6 and 7 and the optically controlled configuration interconversion process of supramolecular complexes. (II) Atomic force microscope (AFM) images of pBR322 DNA (left) and DNA condensation induced by *trans*-7⊂CB[8] (right). Reproduced with permission [187]. Copyright 2014 The Author(s). (**b**) Rodlike supramolecular nanoassemblies for effective delivery of ncRNAs. (I) The synthesis process of supramolecular nanoassemblies and their application for co-delivering pc3.0-MEG3 and pc3.0-miR-101. (II) AFM image of ncRNAs-loaded supramolecular nanoassemblies. (III) Tumor-suppressive effect of ncRNAs-loaded supramolecular nanoassemblies. Reproduced with permission [194]. Copyright 2017 WILEY–VCH Verlag GmbH & Co. KGaA, Weinheim

Molecular machines responding to external stimuli have attracted an increasing number of attentions from different fields [182, 183]. However, adjusting the morphology and functionality of biomolecules by utilizing the reversible shelter of macrocyclic hosts remains challenging [184–186]. Usually, acids and bases are the main driving forces to launch molecular machines, but the physiological environment cannot tolerate strong acids and bases, which guides scientists to the other external stimuli, such as light and heat. Liu et al*.* presented two supramolecular complexes on the basis of host–guest interaction between CB[8], azobenzene and bispyridinium salts (*K*_a_ up to 10^9^ M^_1^), and the dissociation and recombination of which could be reversibly regulated using light and heat (Fig. 9a-I) [187]. Because the positively charged viologen groups in the ‘locked’ configuration draw DNA backbone closely, DNA was tightly condensated (Fig. 9a-II). Nevertheless, the viologen moieties in the ‘unlocked’ configuration were directly exposed to the aqueous solution, which could be activated by UV irradiation to generate ROS and destroy the integrity of DNA. This work provided a new assembly strategy to imitate the collaborative and multipoint binding manners in biological systems.

Gene therapy has developed into a promising strategy to inhibit tumors via delivering versatile tumor-suppressive noncoding RNAs (ncRNAs) [188–191]. Nevertheless, the evolution of gene therapy is impeded by the low transfection efficiency of nonvirus carriers and the safety grounds of virus vectors [192, 193]. Xu et al*.* tailored a supramolecular nanoassembly (CNC@CB[8]@PGEA) equipped with the degradable poly(aspartic acid) (PAsp)-grafted cellulose nanocrystal (CNC) chains and hydroxyl-rich ethanolamine-functionalized poly(glycidyl methacrylate) (PGEA) side chains (Fig. 9b-I) [194]. Attributing to the host–guest self-assembly of CB[8], CNC-PAsp-Np/EA and MV-PGEA, rodlike morphologies were acquired, which combined the unique advantages of CNCs, PAsp and PGEA. CNC@CB[8]@PGEA condensed pc3.0-miR-101 and pc3.0-MEG3 into nanocomplexes with a diameter of about 200 nm (Fig. 9b-II) and implemented the co-transport of short and long ncRNAs in vivo to suppress the growth of hepatocellular carcinoma (HCC) tumor (Fig. 9b-III) without inducing obvious toxicity.

**Fig. 10:**
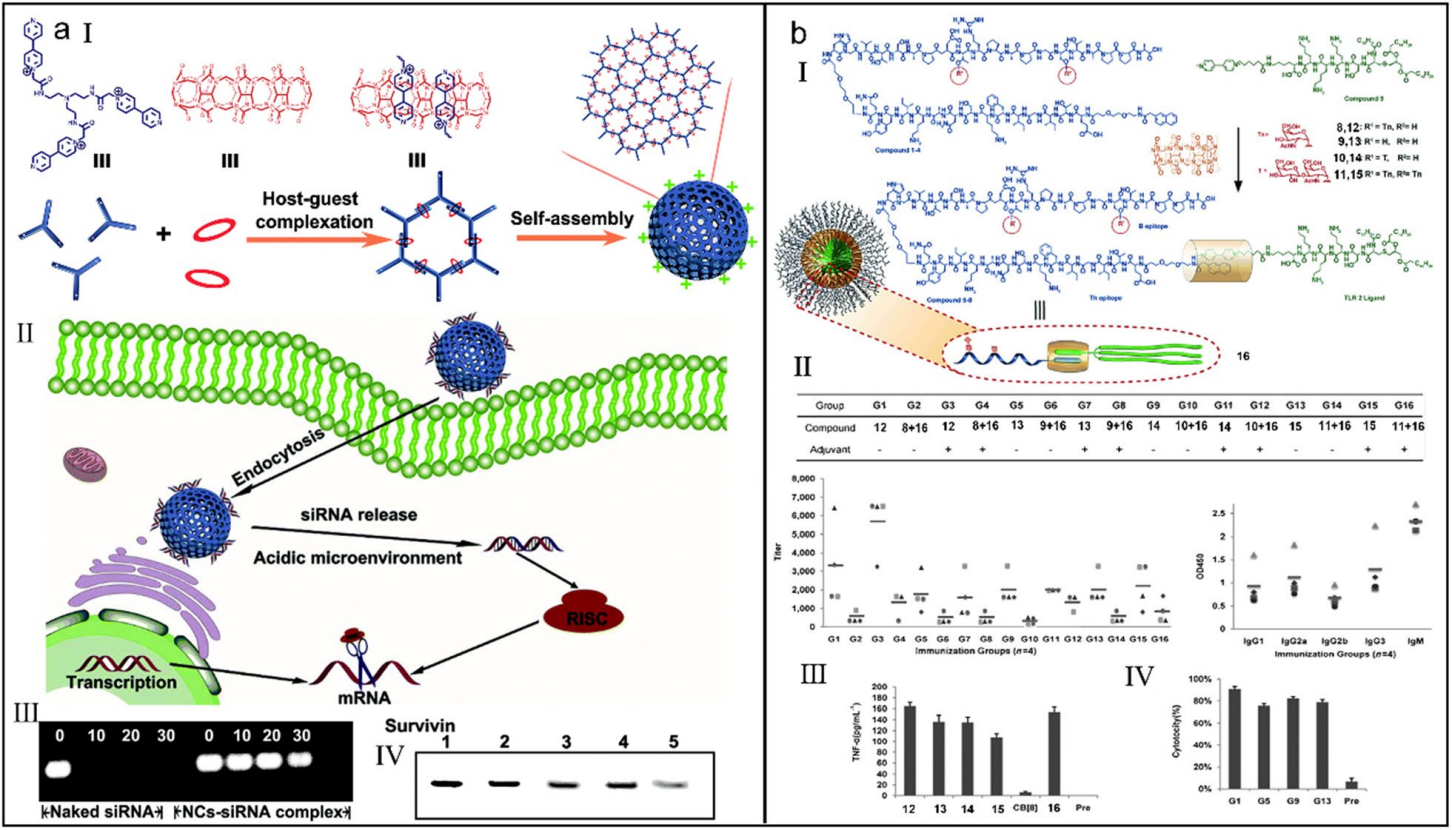
(**a**) Supramolecular polymer nanocapsules for effective siRNA delivery. (I) Illustration of the construction of supramolecular polymer nanocapsules. (II) Illustration of the intracellular siRNA delivery by supramolecular polymer nanocapsules. (III) Biostability test of siRNA with or without supramolecular polymer nanocapsules. (IV) Western blot analysis of intracellular survivin protein after different treatments (1: control; 2: scramble siRNA; 3: lipofectamine 2000-siRNA complex; 4: NC-siRNA complex (50 nM); 5: NC-siRNA complex (100 nM). Reproduced with permission [200]. Copyright 2019 The Royal Society of Chemistry. (**b**) A noncovalent strategy to construct chemically synthesized vaccines. (I) Illustration of the construction of synthesized vaccines. (II) ELISA anti-MUC1 IgG antibody titers (left) and analyses (right) after different immunizations. (III) The secretion of TNF-α cytokine by dendritic cells after different stimulations. (IV) Cytotoxicity assay of MCF-7 cells after different immunizations. Reproduced with permission [206]. Copyright 2014 Wiley–VCH Verlag GmbH & Co. KGaA, Weinheim

Short interfering ribonucleic acid (siRNA) acts as a new hopeful therapeutic agent and has gained significant impetus in tumor therapy [195–199], but weak ribonuclease (RNase) resistance and inefficient cellular uptake greatly limit their therapeutic efficacy and corresponding clinical application. Liu et al*.* constructed a supramolecular nanocapsule (NC) based on the host–guest complexation between a triviologen derivative and CB[8] for siRNA delivery (Fig. 10a-I) [200]. The positive charges on the surface of nanocapsules could bind siRNA and realize intracellular siRNA delivery (Fig. 10a-II). Profiting from the supramolecular self-assembly of nanocapsule, siRNA was protected from enzymatic degradation (Fig. 10a-III) and efficiently suppressed the expression of apoptosis protein (Fig. 10a-IV), suggesting that the established supramolecular nanocapsules serve as an effective siRNA carrier for gene therapy.

Now, chemically synthesized vaccines have abandoned the employment of foreign carrier proteins, thus the original strong B-cell suppressing immune reactions against saccharide and glycopeptide epitopes are weakened [201–205]. With no unnecessary elements, these covalent vaccines have good application foreground, whereas they are hampered by the time-consuming synthesis and characterization. Li et al*.* built a MUC1 glycopeptide antitumor vaccine by using host–guest interaction (Fig. 10b-I) [206]. In detail, different glycosylations acted as the B epitopes, TT830-843 from tetanus toxoid served as the T-helper (Th) cell epitope, and they assembled into the B-epitope–Th-epitope structure. TLR2 ligand Pam_3_CSK_4_ and B-epitope–Th-epitope entity were separately decorated with methyl viologen (MV^2+^) and naphthalene, and they were manacled together by CB[8]. Compared with the simple mixed vaccines, the constructed vaccines elicited a higher level of IgG antibodies (Fig. 10b-II) and cytokine (Fig. 10b-III), and also induced complement-dependent cytotoxicity (Fig. 10b-IV), setting an example for the future chemically synthesized vaccines.

#### Other applications

##### Antimicrobial therapy

Owing to the high mortality and morbidity rate, fungal infection has severely threatened human health [207–210]. Although azoles have developed into the frontline drugs for fungal disease [211], their unprecedented antifungal resistance increases the difficulty of treatment, which in turn drives the development of alternative antifungal therapeutics, such as PDT. Benefiting from the unique twisted structures, aggregation-induced emission (AIE) photosensitizers are always equipped with strong luminous power and high ROS productivity [212]. However, AIE PSs with effective antifungal function often require costly and time-consuming covalent modifications [213], hence developing more promising construction strategy for AIE antifungals is highly needed.

**Fig. 11:**
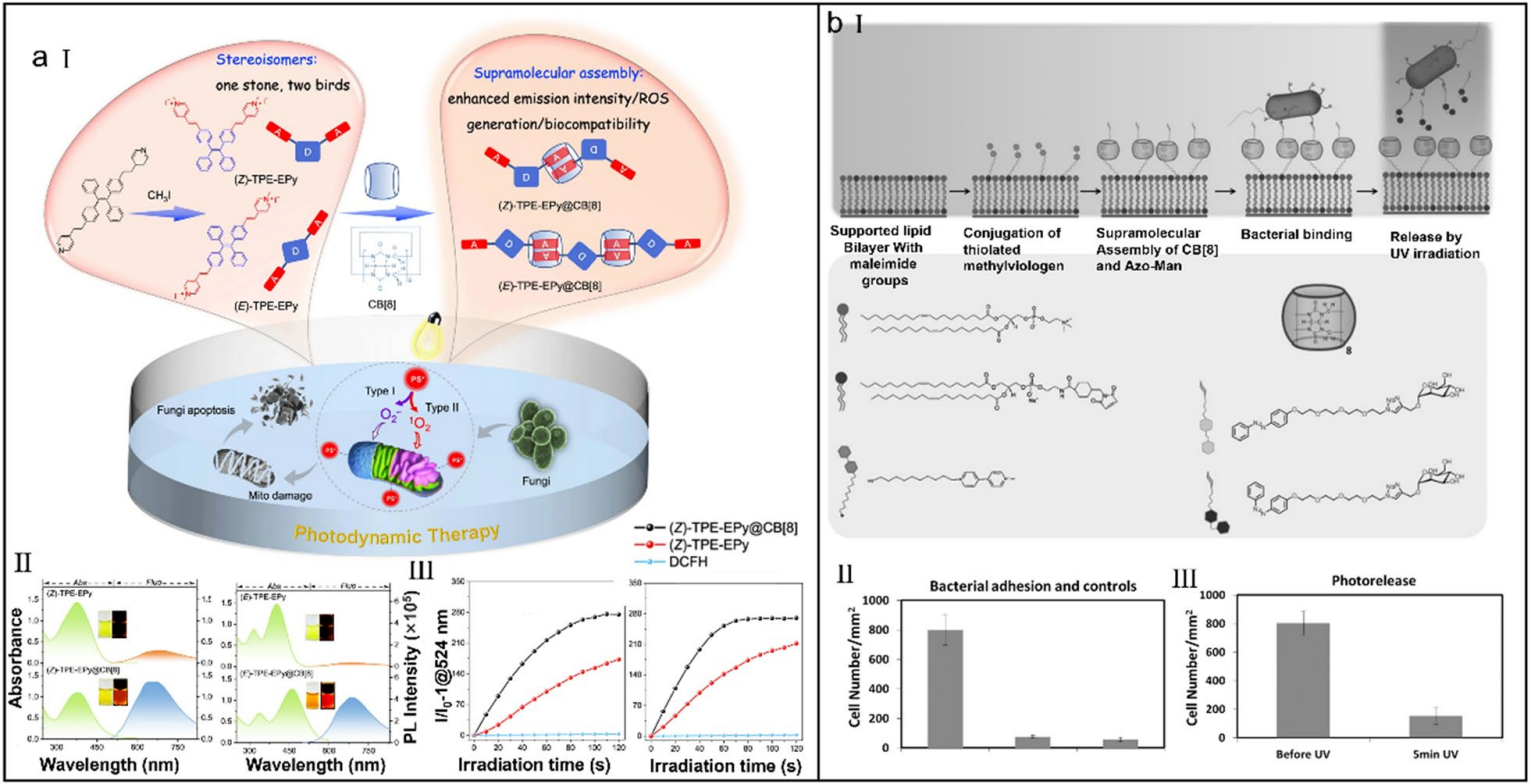
(**a**) Supramolecular engineering of AIE photosensitizers for fungal killing. (I) Chemical structures of stereoisomers and corresponding supramolecular assemblies and the illustration of their sterilization mechanism via PDT. (II) Absorption and emission spectra of stereoisomers. (III) ROS generation assessment of stereoisomers and corresponding supramolecular assemblies. Reproduced with permission [214]. Copyright 2022, The Author(s). (**b**) CB[8]-mediated photoswitchable adhesion and release of bacteria on SLBs. (I) Chemical structures of different components and the illustration of the mechanism of bacteria adhesion and release. (II) The number of bacteria immobilized on supramolecular SLBs. (III) The number of residual bacteria immobilized on supramolecular SLBs. Reproduced with permission [218]. Copyright 2015 Wiley–VCH Verlag GmbH & Co. KGaA, Weinheim

Tang et al*.* developed two stereoisomeric photosensitizers ((Z)/(E)-TPE-EPy) by harnessing host–guest strategies (Fig. 11a-I) [214]. Attributing to the CB[8]-mediated stereoisomeric engineering (*K*_a_ of (*Z*)- and (*E*)-complexs were 5.8 × 10^4^ and 3.6 × 10^5^ M^−1^, respectively), the excited state energy of photosensitizers flowed from the nonradiative decay to the ISC process and radiative decay, which led to the reinforced fluorescence intensity (Fig. 11a-II) and ROS productivity (Fig. 11a-III). Also, electropositivity endowed (Z)/(E)-TPE-EPy with mitochondrial targeting and the targeted antifungal PDT was realized. With the cationic shielding effect of CB[8], the dark toxicity of (Z)/(E)-TPE-EPy@CB[8] was dramatically reduced without sacrificing their PDT efficiency. This supramolecular assembly-assisted stereoisomeric engineering of photosensitizers opens up new doors for combating fungal infections.

Surface immobilization technologies of bioactive ligands have accelerated the development of smart surfaces for biomedical applications [215, 216]. Current surface immobilization strategies ensure the spatial controlling of bioactive ligands [217], but temporal controlling of these ligands needs new strategies. Jonkheijm et al*.* developed the supramolecular supported lipid bilayers (SLBs) based on the supramolecular host–guest chemistry for spatio-temporal release of bacterial cells (Fig. 11b-I) [218]. The photoswitchable supramolecular ternary system was formed by assembling an azobenzene–mannose conjugate (Azo–Man) and CB[8] onto MV^2+^-functionalized liquid-state SLBs. Based on the photo-responsive conformational switching of azobenzene group, *Escherichia coli* (*E. coli*) enabled to bind onto supramolecular SLBs via cell-surface receptors (Fig. 11b-II), and meanwhile was specifically erased by UV irradiation (Fig. 11b-III), thus providing a potential to exploit reusable sensors.

##### Weed control

Because of the simple preparation, reversible oxidation–reduction quality and good electron deficiency, MV^2+^ derivants are the most used guest molecules in the CB[8]-mediated host–guest complexations [46]. In addition to this, MV^2+^ can cut off the electron transport from plastocyanin to nicotinamide adenine dinucleotide phosphate (NADP^+^) and disturb normal functioning of photosystem I (PSI), being able to perform a high herbicidal efficacy in gardening and agriculture [219–221].

**Fig. 12:**
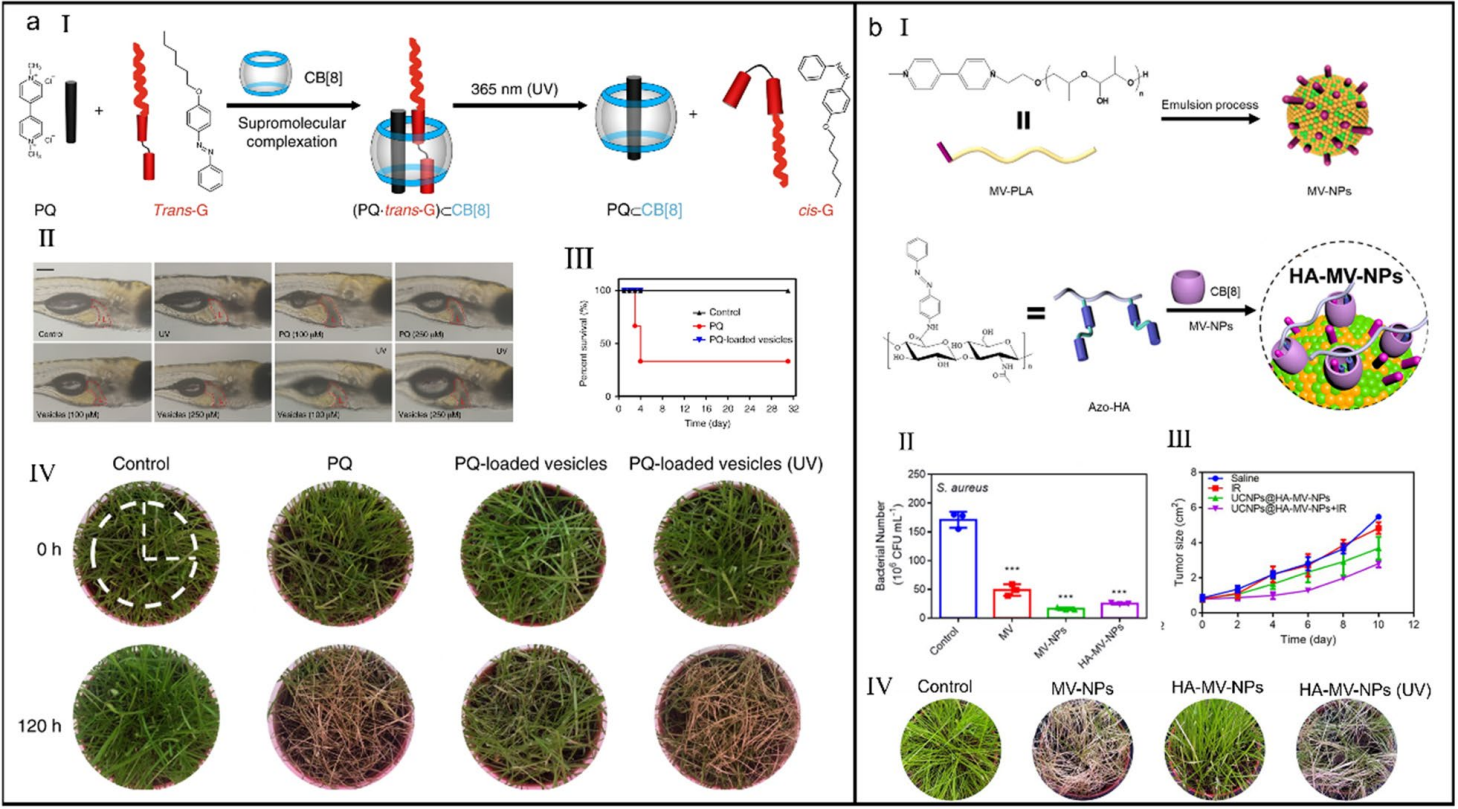
(**a**) Photo-responsive supramolecular vesicles for user-friendly herbicide. (I) Illustration of the CB[8]-mediated supramolecular complexation and photo-driven, reversible complexation and decomplexation. (II) Liver tissue observation of zebrafish after different treatments. (III) Survival curves of mice after different treatments. (IV) Weed control efficacy of different treatment methods. Reproduced with permission [223]. Copyright 2018 The Author(s). (**b**) DIA of supramolecular toxic nanoparticles for multifunctional applications. (I) Illustration of the preparation of MV-NPs and HA-MV-NPs. (II) The comparation of bacteriostasis rate after different treatments. (III) Tumor volume change of mice after different treatments. (IV) Weed control efficacy of different treatment methods. Reproduced with permission [224]. Copyright 2020 American Chemical Society

Nevertheless, since taking a sip can be lethal and there are no valid antidotes clinically available currently, the toxicity of MV^2+^ to humans is always an unsolved safety problem [222]. Wang et al*.* reported a human-friendly, photo-responsive supramolecular herbicide via ternary host–guest self-assembly between an azobenzene derivative (*Trans*-G), MV^2+^ and CB[8] (*K*_a_ = 9.37 (± 2.37) × 10^4^ M^_1^) [223]. Under sunlight or UV irradiation, *Trans*-G converted its configuration from *trans*- to *cis*- form, which in turn dissociated the ternary host–guest interactions and released MV^2+^ to perform herbicidal function (Fig. 12a-I). Due to owning the spatiotemporal controllability, this formulation afforded a safer toxicity profile on both zebrafish (Fig. 12a-II) and murine model (Fig. 12a-III) compared to free MV^2+^. Additionally, the herbicidal activity of supramolecular ternary complex was comparable to that of free MV^2+^ (Fig. 12a-IV), overcoming the safe issue of traditional MV^2+^-loaded antimicrobial agents.

Based on the similar mechanism of ternary host−guest complexation, Wang et al*.* constructed a MV^2+^-sandwiched and HA-coated supramolecular nanoparticles (MV-NPs) for precisely performing bioactivity or toxicity (Fig. 12b-I) [224]. Benefiting by the HA-mediated hyaluronidase (HAase)-responsiveness and azobenzene-guided photo-responsiveness, HA lamination on MV-NPs could be peeled under multiple stimuli such as HAase, UV and IR irradiation, realizing decoating-induced activation (DIA) for selective antibacterial (Fig. 12b-II), anticancer (Fig. 12b-III) and even user-friendly herbicide (Fig. 12b-IV). This work supplied a new supramolecular formulation to tame and control the toxicity and bioactivity of nanomaterials for multifunctional biomedical applications.

##### Biomolecule detection

Immunosuppressive tumor microenvironment is one of the important reasons leading to the failure of tumor therapy, and IDO1 which regulates the metabolism between Trp and F-Kyn, is demonstrated to be an archcriminal for immune escape [225–227]. Therefore, the biocatalytic activity of IDO1 is closely associated with tumor progression. Although various methods have been developed to monitor the expression of IDO1, such as antibody-peptide conjugates, high-performance liquid chromatography (HPLC), colorimetric determination and commercialized Green Screen kit [228–232], but these methods need relatively strict derivatizations that are unsuitable for live cell analysis.

**Fig. 13:**
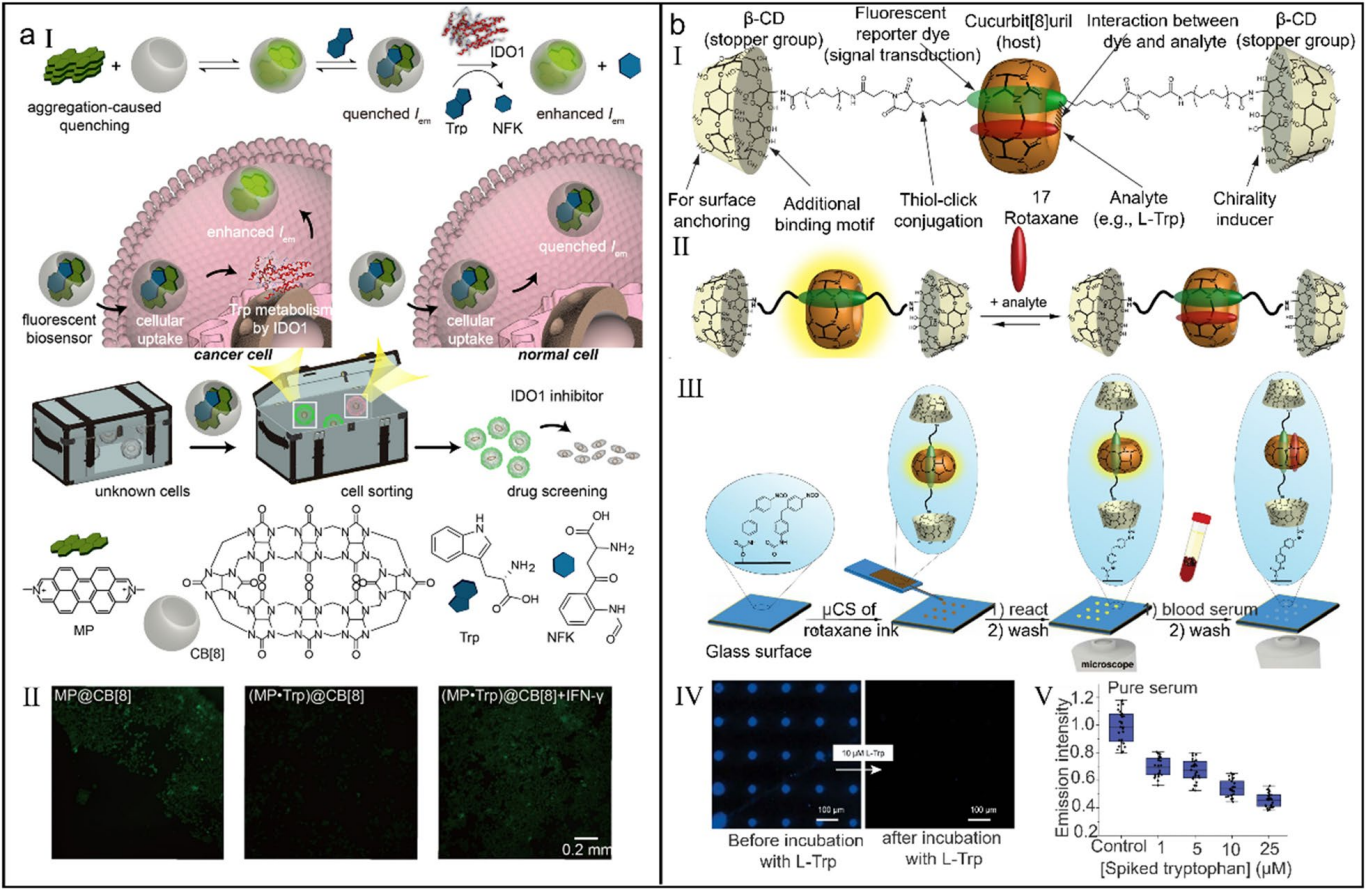
(**a**) An off −on supramolecular fluorescent biosensor for monitoring IDO1 activity in living cells. (I) Illustration of the detection mechanism of supramolecular fluorescent biosensor. (II) Fluorescence images of HepG2 cells with different treatments. Reproduced with permission [233]. Copyright 2019 American Chemical Society. (**b**) CB[8]-based rotaxane chemosensor for optical detection of Trp in biological samples. (I) Design principle of supramolecular rotaxane 17. (II) Illustration of the analyte binding by rotaxane 17. (III) Illustration of the fluorescence imaging of Trp in blood serum by rotaxane 17-immobilizated glass surfaces. (IV) Fluorescence images of a microarray before and after treatment with Trp. (V) Emission intensity change of a sensor chip after treatment with different serums. Reproduced with permission [238]. Copyright 2023 The Author(s)

Hu et al*.* showed a supramolecular tandem method for real-time monitoring the intracellular activity of IDO1 (Fig. 13a-I) [233]. Aggregation-induced quenching dye MP was first encapsulated in the cavity of CB[8] to generate a binary complex MP⊂CB[8] with the enhanced green fluorescence (*K*_a_ > 10^6^ M^_1^), then Trp bound the residual cavity of CB[8] to construct a ternary inclusion (MP·Trp)⊂CB[8], which was accompanied with the complete fluorescence quenching. Once encountering the intracellular IDO1, Trp in complex was immediately oxidized into NFK and luminous MP⊂CB[8] was released to illume cells (Fig. 13a-II). Because IDO1 was overexpressed in tumor cells but not in normal cells and supramolecular sensor was sensitive to the change of intracellular Trp concentration, this label-free method could precisely sort out tumor cells, avoiding the fussy pre-preparation and strict derivatizations.

Although some developed host–guest systems already offer new methods for the inspection of health-relevant biomarker Trp in the complicated media, these systems are usually accompanied with the sophisticated deproteinization and the low sensitivity owing to their weak binding affinities with Trp [234–237], thus realizing the accurate detection of Trp in untreated biological samples is highly pursued. Biedermann et al*.* constructed a rotaxane chemosensor for direct detection of Trp in blood and urine samples, in which CB[8], a reporter dye and β-CD respectively acted as macrocyclic molecule, axial component and stopper group (Fig. 13b-I) (log *K*_a_ = 0.2) [238]. Upon Trp drilling into the cavity of CB[8], a face-to-face π–π stacking occurred between electron-deficient dye and electron-rich Trp, which induced the charge-transfer interactions and significantly quenched the fluorescence of the reporter dye (Fig. 13b-II). This supramolecular chemosensor not only enabled high-throughput screen in a microwell plate but also realized chirality sensing and label-free enzyme reaction monitoring. Moreover, printed sensor chips outwardly immobilized with the rotaxane-microarrays could be used for fluorescence imaging of Trp (Fig. 13b-III–V), greatly overcoming the limitations of sensing in biofluids and inspiring the development of new supramolecular chemosensors for molecular diagnostics.

**Fig. 14:**
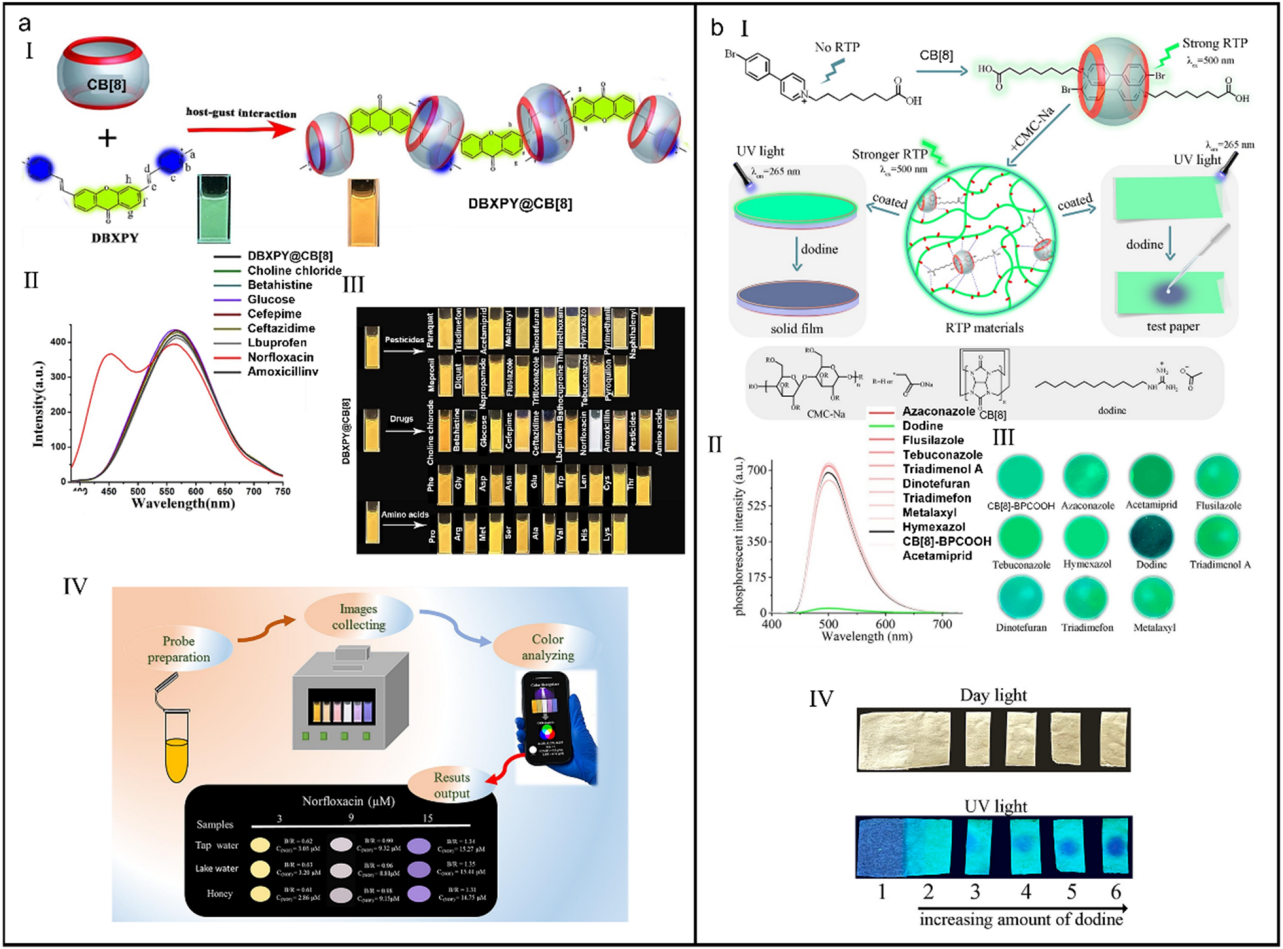
(**a**) A supramolecular fluorescent probe for determination of norfloxacin. (I) Schematic illustration of the self-assembly of supramolecular fluorescent probe. (II) The fluorescence emission change of DBXPY@CB[8] after addition of different drugs. (III) Fluorescence photographs of DBXPY@CB[8] after addition of various drugs, pesticides and amino acids. (IV) Schematic illustration of the detection process of supramolecular fluorescent probe. Reproduced with permission [239]. Copyright 2023 Elsevier B.V. All rights reserved. (**b**) Supramolecular phosphorescent probe for determination of dodine. (I) Chemical structures of different components and the schematic illustration of the detection mechanism of supramolecular phosphorescent probe. (II) Phosphorescent emission change of CB[8]-BPCOOH after addition of different pesticides. (III) Phosphorescent photographs of CB[8]-BPCOOH-based solid film in the presence of different pesticides. (IV) Phosphorescent photographs of CB[8]-BPCOOH-based indicator paper in the presence of different concentrations of dodine. Reproduced with permission [240]. Copyright 2022 American Chemical Society

Norfloxacin (NOF), a third generation of quinolone antibiotics, has been widely used in the daily life of people. Whereas, the overuse of NOF has meanwhile caused serious environmental pollution as it has been detected in soil, surface water and even groundwater and drinking water. To date, several analytical methods including HPLC, side-flow immunoassay strip (LFIS), ELISA, surface-enhanced Raman spectroscopy (SERS) and capillary electrophoresis (CE) have been used to detect NOF, but expensive and time-consuming pretreatment and professional analysis technics are needed. Xiao et al*.* reported a supramolecular fluorescence probe (DBXPY@CB[8]) to rapidly and sensitively detect norfloxacin based on host–guest interaction between CB[8] and dibromoxanthen-9-one phenylpyridine cationic derivative (DBXPY) (Fig. 14a-I) [239]. The addition of norfloxacin induced an obvious blue-shift of DBXPY@CB[8], and the detection of NOF was not affected by pesticides, amino acids and other antibiotics which contributed to a low detection limit (1.08 × 10^−7^ M) (Fig. 14a-II and III). With the help of smart phone RGB analysis, a quantitative and visual detection of norfloxacin in food and water can be realized without any precision instrument (Fig. 14a-IV), performing a great improvement over conventional techniques.

Different from the above fluorescence detections, Xiao et al*.* developed a supramolecular charge-transfer dimer (CB[8]-BPCOOH) featuring RTP for detection of dodine (Fig. 14b-I) [240]. Benefiting by the host–guest interaction between CB[8] and BPCOOH, the molecular rotation of BPCOOH was inhibited and the water molecules and oxygen in surrounding microenvironment were isolated, which significantly improved the RTP emission behavior of BPCOOH. Interestingly, CB[8]-BPCOOH only specifically recognized dodine among other 10 pesticides (Fig. 14b-II), performing a dual detection capacity (phosphorescence quenching and meanwhile fluorescence enhancing), thus greatly improving the detection accuracy. Furthermore, CB[8]-BPCOOH could be functionalized into solid films (Fig. 14b-III) and indicator papers (Fig. 14b-IV) which were equipped with the advantages of fast identification and easy portability, providing more probabilities for cucurbit[*n*]uril-based RTP material.

## Conclusion and outlooks

Now, a myriad of CB[8]-based supramolecular theranostic systems have been developed to improve the limitations of current medical technologies. Benefiting from the CB[8]-based host–guest chemistry, the solubility/stability, pharmacokinetics behaviors as well as the duration of activity of loaded-drugs are significantly improved, hopefully fulfilling the high requirements of personalized treatment. Owing to the “Lego-like” self-assembly modes and dynamic reversibility of host–guest chemistry, not only the synthesis and purification is easy and feasible, but also the spatial and temporal drug release can be realized, greatly enriching the theranostic functions and reducing the side effects. Despite CB[8]-based supramolecular theranostics have been vastly developed and acquired a great deal of brilliant progresses over the past years, there are still irremissible issues to be overcame.

- Compared to cyclodextrins with a good commercial availability in various sizes, CB[8] is not at an affordable cost nor commercially available on a large scale, which has hindered its applications in the field of pharmaceutical science communities and biomaterials. Therefore, this challenge requires continuous concerted efforts from synthetic chemists, pharmacist, and biologists to optimize the preparation conditions for the large-scale preparation of CB[8].
- Owing to the weak solubility both in water and organic solvents, CB[8] is quite chemically inert and its functionalization becomes a daunting task as a consequence. Considering the developments brought by CB[8] in the field of biomedical, there is no doubt that a number of possibilities remains to be explored in case that the functionalization of CB[8] can be unlocked.
- Except for cyclodextrins, almost no macrocycles including CB[8], have been approved or even used in clinical practice owing to their potential biotoxicity and immunogenicity. More attentions should be paid to the biocompatibility and degradability of CB[8] to avoid the systemic toxicity and immunotoxicities.
- Although CB[8]-based supramolecular theranostic systems have been engaged in a variety of biomedical fields, as mentioned and referenced earlier in this Review, more complicated theranostic means are not involved, such as ultrasound imaging (US), photoacoustic imaging (PA), single-photon emission computed tomography (SPECT), magnetic resonance imaging (MRI), X-ray computed tomography (CT), radiotherapy, ultrasound therapy and smart immunotherapy. It is the high time to develop more novel supramolecular theranostics via reasonably crossing chemistry, pharmacology, materials engineering, cancer biology and oncology.

In conclusion, we passionately believe that CB[8] and their derivatives are highly promising and potent candidates in constructing smart supramolecular nanotheranostics with the improved therapeutic effects. Prominent improvement and achievements will be achieved in the field of supramolecular theranostics and meaningful improvement of health services of human beings will be observed benefiting from the intelligent development of CB[8]-based biomaterials in the near future.

## References

[1] Song N, Lou X-Y, Ma L, Gao H, Yang Y-W (2019). Supramolecular nanotheranostics based on pillarenes. Theranostics.

[2] Gravel J, Schmitzer AR (2017). Imidazolium and benzimidazolium-containing compounds: from simple toxic salts to highly bioactive drugs. Org Biomol Chem.

[3] Karimi M, Zangabad PS, Mehdizadeh F, Malekzad H, Ghasemi A, Bahrami S, Zare H, Moghoofei M, Hekmatmanesh A, Hamblin MR (2017). Nanocaged platforms: modification, drug delivery and nanotoxicity. Opening synthetic cages to release the tiger. Nanoscale.

[4] Wu D, Zhang Z, Li X, Zhu T, Wang J, Hu Q (2023). Supramolecular theranostic nanomedicine for in situ self-boosting cancer photochemotherapy. Biomacromol.

[5] Wu D, Zhang Z, Li X, Han J, Hu Q, Yu Y, Mao Z (2023). Cucurbit[10]uril-based supramolecular radicals: powerful arms to kill facultative anaerobic bacteria. J Control Release.

[6] Wu D, Zhang Z, Li X, Zhou J, Cao Y, Qi S, Wang L, Liu Z, Yu G (2023). Dynamically assembled nanomedicine based on host–guest molecular recognition for NIR laser-excited chemotherapy and phototheranostics. Acta Biomater.

[7] Song N, Yang Y-W (2015). Molecular and supramolecular switches on mesoporous silica nanoparticles. Chem Soc Rev.

[8] Wen J, Yang K, Liu F, Li H, Xu Y, Sun S (2017). Diverse gatekeepers for mesoporous silica nanoparticle based drug delivery systems. Chem Soc Rev.

[9] Nguyen KT, Zhao Y (2015). Engineered hybrid nanoparticles for on-demand diagnostics and therapeutics. Acc Chem Res.

[10] Yang Y-W, Sun Y-L, Song N (2014). Switchable host–guest systems on surfaces. Acc Chem Res.

[11] Zhou J, Rao L, Yu G, Cook TR, Chen X, Huang F (2021). Supramolecular cancer nanotheranostics. Chem Soc Rev.

[12] Zhou J, Yu G, Huang F (2017). Supramolecular chemotherapy based on host–guest molecular recognition: a novel strategy in the battle against cancer with a bright future. Chem Soc Rev.

[13] Yan M, Wu S, Wang Y, Liang M, Wang M, Hu W, Yu G, Mao Z, Huang F, Zhou J (2023). Recent progress of supramolecular chemotherapy based on host–guest interactions. Adv Mater.

[14] Yu G, Chen X (2019). Host-guest chemistry in supramolecular theranostics. Theranostics.

[15] Chen X, Zheng G, Cheng J, Yang Y-Y (2019). Supramolecular nanotheranostics. Theranostics.

[16] Wang L, Li L-L, Fan Y-S, Wang H (2013). Host–guest supramolecular nanosystems for cancer diagnostics and therapeutics. Adv Mater.

[17] Wu H, Chen Z, Qi S, Bai B, Ye J, Wu D, Shen J, Kang F, Yu G (2021). Evaluation of the stability of cucurbit[8]uril-based ternary host−guest complexation in physiological environment and the fabrication of a supramolecular theranostic nanomedicine. J Nanobiotechnol.

[18] Yuan M, Chen T, Jin L, Zhang P, Xie L, Zhou S, Fan L, Wang L, Zhang C, Tang N, Guo L, Xie C, Duo Y, Li L, Shi L (2023). A carrier-free supramolecular nano-twin-drug for overcoming irinotecan-resistance and enhancing efficacy against colorectal cancer. J Nanobiotechnol.

[19] Zhou J, Zhang Y, Yu G, Crawley MR, Fulong CRP, Friedman AE, Sengupta S, Sun J, Li Q, Huang F, Cook TR (2018). Highly emissive self-assembled BODIPY-platinum supramolecular triangles. J Am Chem Soc.

[20] Liu J, Chen C, Wei T, Gayet O, Loncle C, Borge L, Dusetti N, Ma X, Marson D, Laurini E, Pricl S, Gu Z, Iovanna J, Peng L, Liang X-J (2021). Dendrimeric nanosystem consistently circumvents heterogeneous drug response and resistance in pancreatic cancer. Exploration.

[21] Wu S, Yan M, Liang M, Yang W, Chen J, Zhou J (2023). Supramolecular host–guest nanosystems for overcoming cancer drug resistance. Cancer Drug Resist.

[22] Yao X, Yang B, Xu J, He Q, Yang W (2022). Novel gas-based nanomedicines for cancer therapy. VIEW.

[23] Wu N, Tu Y, Fan G, Ding J, Luo J, Wang W, Zhang C, Yuan C, Zhang H, Chen P, Tan S, Xiao H (2022). Enhanced photodynamic therapy/photothermo therapy for nasopharyngeal carcinoma via a tumour microenvironment-responsive self-oxygenated drug delivery system. Asian J Pharm Sci.

[24] Ding Y, Tong Z, Jin L, Ye B, Zhou J, Sun Z, Yang H, Hong L, Huang F, Wang W, Mao Z (2022). An NIR discrete metallacycle constructed from perylene bisimide and tetraphenylethylene fluorophores for imaging-guided cancer radio-chemotherapy. Adv Mater.

[25] Yan M, Zhou J (2023). Pillararene-based supramolecular polymers for cancer therapy. Molecules.

[26] Yan M, Zhou J (2023). Suprasomes: an emerging platform for cancer theranostics. Sci China Chem.

[27] Cafeo G, Carbotti G, Cuzzola A, Fabbi M, Ferrini S, Kohnke FH, Papanikolaou G, Plutino MR, Rosano C, White AJP (2013). Drug delivery with a calixpyrrole–trans-Pt(II) complex. J Am Chem Soc.

[28] Challa R, Ahuja A, Ali J, Khar RK (2005). Cyclodextrins in drug delivery: an updated review. AAPS Pharm Sci Tech.

[29] Laza-Knoerr AL, Gref R, Couvreur P (2010). Cyclodextrins for drug delivery. J Drug Target.

[30] Tibbitt MW, Dahlman JE, Langer R (2016). Emerging frontiers in drug delivery. J Am Chem Soc.

[31] Yu G, Yung BC, Zhou Z, Mao Z, Chen X (2018). Artificial molecular machines in nanotheranostics. ACS Nano.

[32] Webber MJ, Langer R (2017). Drug delivery by supramolecular design. Chem Soc Rev.

[33] Webber MJ, Appel EA, Meijer EW, Langer R (2016). Supramolecular biomaterials. Nat Mater.

[34] Zhang J, Ma PX (2013). Cyclodextrin-based supramolecular systems for drug delivery: recent progress and future perspective. Adv Drug Deliv Rev.

[35] Cabral H, Nishiyama N, Kataoka K (2011). Supramolecular nanodevices: from design validation to theranostic nanomedicine. Acc Chem Res.

[36] Ma X, Zhao Y (2015). Biomedical applications of supramolecular systems based on host–guest interactions. Chem Rev.

[37] Peng L, Liu S, Feng A, Yuan J (2017). Polymeric nanocarriers based on cyclodextrins for drug delivery: host–guest interaction as stimuli responsive linker. Mol Pharm.

[38] Tu Z, Guday G, Adeli M, Haag R (2018). Multivalent interactions between 2D nanomaterials and biointerfaces. Adv Mater.

[39] Yu G, Zhang M, Saha ML, Mao Z, Chen J, Yao Y, Zhou Z, Liu Y, Gao C, Huang F, Chen X, Stang PJ (2017). Antitumor activity of a unique polymer that incorporates a fluorescent self-assembled metallacycle. J Am Chem Soc.

[40] Kim J, Jung I-S, Kim S-Y, Lee E, Kang J-K, Sakamoto S, Yamaguchi K, Kim K (2000). New cucurbituril homologues: syntheses, isolation, characterization, and X-ray crystal structures of cucurbit-[n]uril (n= 5, 7, and 8). J Am Chem Soc.

[41] Day A, Arnold AP, Blanch RJ, Snushall B (2001). Controlling factors in the synthesis of cucurbituril and its homologues. J Org Chem.

[42] Day AI, Blanch RJ, Arnold AP, Lorenzo S, Lewis GR, Dance IA (2002). Cucurbituril-based gyroscane: a new supramolecular form. Angew Chem Int Ed.

[43] Cheng X-J, Liang L-L, Chen K, Ji N-N, Xiao X, Zhang J-X, Zhang Y-Q, Xue S-F, Zhu Q-J, Ni X-L, Tao Z (2013). Twisted cucurbit[14]uril. Angew Chem Int Ed.

[44] Li Q, Qiu S-C, Zhang J, Chen K, Huang Y, Xiao X, Zhang Y, Li F, Zhang Y-Q, Xue S-F, Zhu Q-J, Tao Z, Lindoy LF, Wei G (2016). Twisted cucurbit[n]urils. Org Lett.

[45] Francisco V, Lino M, Ferreira L (2019). A near infrared light-triggerable modular formulation for the delivery of small biomolecules. J Nanobiotechnol.

[46] Cao L, Śekutor M, Zavalij PY, Mlinarić-Majerski K, Glaser R, Isaacs L (2014). Cucurbit[7]uril⋅guest pair with an attomolar dissociation constant. Angew Chem Int Ed Engl.

[47] Lagona J, Mukhopadhyay P, Chakrabarti S, Isaacs L (2005). The cucurbit[n]uril family. Angew Chem Int Ed.

[48] Lee JW, Samal S, Selvapalam N, Kim H-J, Kim K (2003). Cucurbituril homologues and derivatives: new opportunities in supramolecular chemistry. Acc Chem Res.

[49] Masson E, Ling X, Joseph R, Kyeremeh-Mensah L, Lu X (2012). Cucurbituril chemistry: a tale of supramolecular success. RSC Adv.

[50] Isaacs L (2014). Stimuli responsive systems constructed using cucurbit[n]uril-type molecular containers. Acc Chem Res.

[51] Liu J, Lan Y, Yu Z, Tan CSY, Parker RM, Abell C, Scherman OA (2017). Cucurbit[n]uril-based microcapsules self-assembled within microfluidic droplets: a versatile approach for supra-molecular architectures and materials. Acc Chem Res.

[52] Kaifer AE (2014). Toward reversible control of cucurbit[n]uril complexes. Acc Chem Res.

[53] Barrow SJ, Kasera S, Rowland MJ, del Barrio J, Scherman OA (2015). Cucurbituril-based molecular recognition. Chem Rev.

[54] Assaf KI, Nau WM (2015). Cucurbiturils: from synthesis to high-affinity binding and catalysis. Chem Soc Rev.

[55] Lü J, Lin J-X, Cao M-N, Cao R (2013). Cucurbituril: a promising organic building block for the design of coordination compounds and beyond. Coord Chem Rev.

[56] Yang D, Liu M, Xiao X, Tao Z, Redshaw C (2021). Polymeric self-assembled cucurbit[n]urils: synthesis, structures and applications. Coord Chem Rev.

[57] Lin R-L, Liu J-X, Chen K, Redshaw C (2020). Supramolecular chemistry of substituted cucurbit[n]urils. Inorg Chem Front.

[58] Zhang X-D, Chen K, Sun W-Y (2021). Potential applications of cucurbit[n]urils and their derivatives in the capture of hazardous chemicals. Chem Eur J.

[59] Kim H-J, Heo J, Jeon WS, Lee E, Kim J, Sakamoto S, Yamaguchi K, Kim K (2001). Selective inclusion of a hetero-guest pair in a molecular host: formation of stable charge-transfer complexes in cucurbit[8]uril. Angew Chem Int Ed Engl.

[60] Kim H-J, Jeon WS, Ko YH, Kim K (2002). Inclusion of methylviologen in cucurbit[7]uril. Proc Natl Acad Sci USA.

[61] Nally R, Scherman OA, Isaacs L (2010). Polymer deaggregation and assembly controlled by a double cavity cucurbituril. Supramol Chem.

[62] Appel EA, del Barrio J, Dyson J, Isaacs L, Scherman OA (2012). Metastable single-chain polymernanoparticles prepared by dynamic cross-linking with nor-seco-cucurbit[10]uril. Chem Sci.

[63] Park KM, Roh JH, Sung G, Murray J, Kim K (2017). Self-healable supramolecular hydrogel formed by nor-seco-cucurbit[10]uril as a supramolecular crosslinker. Chem Asian J.

[64] Wang Z, Shui M, Wyman IW, Zhang Q-W, Wang R (2021). Cucurbit[8]uril-based supramolecular hydrogels for biomedical applications. RSC Med Chem.

[65] Elena Pazos, Novo P, Peinador C, Kaifer AE, García MD (2019). Cucurbit[8]uril (CB[8])-based supramolecular switches. Angew Chem Int Ed Engl.

[66] Zou H, Liu J, Li Y, Li X, Wang X (2018). Cucurbit[8]uril-based polymers and polymer materials. Small.

[67] Wang Z, Sun C, Yang K, Chen X, Wang R (2022). Cucurbituril-based supramolecular polymers for biomedical applications. Angew Chem Int Ed Engl.

[68] Huang Y, Gao R-H, Liu M, Chen L-X, Ni X-L, Xiao X, Cong H, Zhu Q-J, Chen K, Tao Z (2021). Cucurbit[n]uril-based supramolecular frameworks assembled through outer-surface interactions. Angew Chem Int Ed Engl.

[69] Park KM, Hur MY, Ghosh SK, Boraste DR, Kim S, Kim K (2019). Cucurbit[n]uril-based amphiphiles that self-assemble into functional nanomaterials for therapeutics. Chem Commun.

[70] Liu J-X, Chen K, Redshaw C (2023). Stimuli-responsive mechanically interlocked molecules constructed from cucurbit[n]uril homologues and derivatives. Chem Soc Rev.

[71] Nie H, Wei Z, Ni X-L, Liu Y (2022). Assembly and applications of macrocyclic-confinement-derived supramolecular organic luminescent emissions from cucurbiturils. Chem Rev.

[72] Anees P, Sreejith S, Ajayaghosh A (2014). Self-assembled near-infrared dye nanoparticles as a selective protein sensor by activation of a dormant fluorophore. J Am Chem Soc.

[73] Hu X, Hu J, Tian J, Ge Z, Zhang G, Luo K, Liu S (2013). Polyprodrug amphiphiles: hierarchical assemblies for shape-regulated cellular internalization, trafficking, and drug delivery. J Am Chem Soc.

[74] Shi B, Jie K, Zhou Y, Zhou J, Xia D, Huang F (2016). Nanoparticles with near-infrared emission enhanced by pillararene-based molecular recognition in water. J Am Chem Soc.

[75] Weissleder R, Pittet MJ (2008). Imaging in the era of molecular oncology. Nature.

[76] Liu Y, Chen M, Cao T, Sun Y, Li C, Liu Q, Yang T, Yao L, Feng W, Li F (2013). A cyanine-modified nanosystem for in vivo upconversion luminescence bioimaging of methylmercury. J Am Chem Soc.

[77] Sun C, Lu J, Wang J, Hao P, Li C, Qi L, Yang L, He B, Zhong Z, Hao N (2021). Redox-sensitive polymeric micelles with aggregation-induced emission for bioimaging and delivery of anticancer drugs. J Nanobiotechnol.

[78] Chen X-M, Chen Y, Yu Q, Gu B-H, Liu Y (2018). Supramolecular assemblies with near-infrared emission mediated in two stages by cucurbituril and amphiphilic calixarene for lysosome-targeted cell imaging. Angew Chem Int Ed Engl.

[79] Dai D, Li Z, Yang J, Wang C, Wu J-R, Wang Y, Zhang D, Yang Y-W (2019). Supramolecular assembly-induced emission enhancement for efficient mercury(II) detection and removal. J Am Chem Soc.

[80] Li Y, Dong Y, Miao X, Ren Y, Zhang B, Wang P, Yu Y, Li B, Isaacs L, Cao L (2018). Shape-controllable and fluorescent supramolecular organic frameworks through aqueous host–guest complexation. Angew Chem Int Ed.

[81] Liu Z, Dai X, Sun Y, Liu Y (2020). Organic supramolecular aggregates based on water-soluble cyclodextrins and calixarenes. Aggregate.

[82] Li J, Wang J, Li H, Song N, Wang D, Tang B (2020). Supramolecular materials based on AIE luminogens (AIEgens): construction and applications. Chem Soc Rev.

[83] Mako TL, Racicot JM, Levine M (2019). Supramolecular luminescent sensors. Chem Rev.

[84] Shen F-F, Zhang Y-M, Dai X-Y, Zhang H-Y, Liu Y (2020). Alkyl-substituted cucurbit[6]uril bridged β-cyclodextrin dimer mediated intramolecular FRET behavior. J Org Chem.

[85] Pawlicki M, Collins HA, Denning RG, Anderson HL (2009). Two-photon absorption and the design of two-photon dyes. Angew Chem Int Ed.

[86] Wu L, Liu J, Li P, Tang B, James TD (2021). Two-photon small-molecule fluorescence-based agents for sensing, imaging, and therapy within biological systems. Chem Soc Rev.

[87] Zheng Z, Li D, Liu Z, Peng H-Q, Sung HHY, Kwok RTK, Williams ID, Lam JWY, Qian J, Tang B (2019). Aggregation-induced nonlinear optical effects of AIEgen nanocrystals for ultradeep in vivo bioimaging. Adv Mater.

[88] Cai X, Wang K-N, Ma W, Yang Y, Chen G, Fu H, Cui C, Yu Z, Wang X (2021). Multifunctional AIE iridium (III) photosensitizer nanoparticles for two-photon-activated imaging and mitochondria targeting photodynamic therapy. J Nanobiotechnol.

[89] Duan W, Li B, Zhang W, Li J, Yao X, Tian Y, Zheng J, Li D (2022). Two-photon responsive porphyrinic metal-organic framework involving Fenton-like reaction for enhanced photodynamic and sonodynamic therapy. J Nanobiotechnol.

[90] Shen F-F, Chen Y, Xu X, Yu H-J, Wang H, Liu Y (2021). Supramolecular assembly with near-infrared emission for two-photon mitochondrial targeted imaging. Small.

[91] Sicard LJ, Li H-C, Wang Q, Liu X-Y, Jeannin O, Rault-Berthelot J, Liao L-S, Jiang Z-Q, Poriel C (2019). C1-linked spirobifluorene dimers: pure hydrocarbon hosts for high-performance blue phosphorescent OLEDs. Angew Chem Int Ed.

[92] Kimura K, Miwa K, Imada H, Imai-Imada M, Kawahara S, Takeya J, Kawai M, Galperin M, Kim Y (2019). Selective triplet exciton formation in a single molecule. Nature.

[93] Lu C, Su Q, Yang X (2019). Ultra-long room-temperature phosphorescent carbon dots: pH sensing and dual-channel detection of tetracyclines. Nanoscale.

[94] Zhou Y, Qin W, Du C, Gao H, Zhu F, Liang G (2019). Long-lived room-temperature phosphorescence for visual and quantitative detection of oxygen. Angew Chem Int Ed.

[95] Wang Y, Gao H, Yang J, Fang M, Ding D, Tang B, Li Z (2021). High performance of simple organic phosphorescence host–guest materials and their application in time-resolved bioimaging. Adv Mater.

[96] Zhao B, Wang H, Xie M, Han C, Yang H, Zhao W, Zhao Q, Xu H (2020). Phosphine oxides manipulate aggregation-induced delayed fluorescence for time-resolved bioimaging. Adv Photonics Res.

[97] Zhao B, Xie G, Wang H, Han C, Xu H (2019). Simply structured near-infrared emitters with a multicyano linear acceptor for solution-processed organic light-emitting diodes. Chem Eur J.

[98] Wang J, Huang Z, Ma X, Tian H (2020). Visible-light-excited room-temperature phosphorescence in water by cucurbit[8]uril-mediated supramolecular assembly. Angew Chem Int Ed Engl.

[99] Huo M, Dai X-Y, Liu Y (2022). Uncommon supramolecular phosphorescence-capturing assembly based on cucurbit[8]uril-mediated molecular folding for near-infrared lysosome imaging. Small.

[100] Xing W-W, Wang H-J, Liu Z, Yu Z-H, Zhang H-Y, Liu Y (2023). Photoreaction boosting phosphorescence cascade energy transfer based on cucurbit[8]uril biaxial polypseudorotaxane. Adv Optical Mater.

[101] Zhang Y-M, Liu Y-H, Liu Y (2020). Cyclodextrin-based multistimuli-responsive supramolecular assemblies and their biological functions. Adv Mater.

[102] Cook AB, Decuzzi P (2021). Harnessing endogenous stimuli for responsive materials in theranostics. ACS Nano.

[103] Lu Y, Aimetti AA, Langer R, Gu Z (2016). Bioresponsive materials. Nat Rev Mater.

[104] Luo C, Sun J, Liu D, Sun B, Miao L, Musetti S, Li J, Han X, Du Y, Li L, Huang L, He Z (2016). Self-assembled redox dual-responsive prodrug-nanosystem formed by single thioether-bridged paclitaxel-fatty acid conjugate for cancer chemotherapy. Nano Lett.

[105] Wang Y, Shim MS, Levinson NS, Sung HW, Xia Y (2014). Stimuli-responsive materials for controlled release of theranostic agents. Adv Funct Mater.

[106] Liu Y-H, Tang M, Zhou X, Liu Y (2022). Biaxial pseudorotaxane secondary assembly for phosphorescent cellular imaging. Mater Adv.

[107] Cai S, Shi H, Li J, Gu L, Ni Y, Cheng Z, Wang S, Xiong W-W, Li L, An Z, Huang W (2017). Visible-light-excited ultralong organic phosphorescence by manipulating intermolecular interactions. Adv Mater.

[108] Wang X-F, Xiao H, Chen P-Z, Yang Q-Z, Chen B, Tung C-H, Chen Y-Z, Wu L-Z (2019). Pure organic room temperature phosphorescence from excited dimers in self-assembled nanoparticles under visible and near-infrared irradiation in water. J Am Chem Soc.

[109] Wang S, Gu K, Guo Z, Yan C, Yang T, Chen Z, Tian H, Zhu W-H (2019). Self-assembly of a monochromophore-based polymer enables unprecedented ratiometric tracing of hypoxia. Adv Mater.

[110] Zhou W-L, Chen Y, Yu Q, Zhang H, Liu Z-X, Dai X-Y, Li J-J, Liu Y (2020). Ultralong purely organic aqueous phosphorescence supramolecular polymer for targeted tumor cell imaging. Nat Commun.

[111] Cabral H, Matsumoto Y, Mizuno K, Chen Q, Murakami M, Kimura M, Terada Y, Kano MR, Miyazono K, Uesaka M, Nishiyama N, Kataoka K (2011). Accumulation of sub-100 nm polymeric micelles in poorly permeable tumours depends on size. Nat Nanotechnol.

[112] Devadasu VR, Bhardwaj V, Kumar MNVR (2013). Can controversial nanotechnology promise drug delivery?. Chem Rev.

[113] Veiseh O, Tang BC, Whitehead KA, Anderson DG, Langer R (2015). Managing diabetes with nanomedicine: challenges and opportunities. Nat Rev Drug Discovery.

[114] Min Y, Caster JM, Eblan MJ, Wang AZ (2015). Clinical translation of nanomedicine. Chem Rev.

[115] Wu W, Pu Y, Shi J (2022). Nanomedicine-enabled chemotherapy-based synergetic cancer treatments. J Nanobiotechnol.

[116] Doane TL, Burda C (2012). The unique role of nanoparticles in nanomedicine: imaging, drug delivery and therapy. Chem Soc Rev.

[117] Mura S, Nicolas J, Couvreur P (2013). Stimuli-responsive nanocarriers for drug delivery. Nat Mater.

[118] Blanco E, Shen H, Ferrari M (2015). Principles of nanoparticle design for overcoming biological barriers to drug delivery. Nat Biotechnol.

[119] Huynh E, Leung BYC, Helfield BL, Shakiba M, Gandier J-A, Jin CS, Master ER, Wilson BC, Goertz DE, Zheng G (2015). In situ conversion of porphyrin microbubbles to nanoparticles for multimodality imaging. Nat Nanotechnol.

[120] Wu D, Li Y, Yang J, Shen J, Zhou J, Hu Q, Yu G, Tang G, Chen X (2017). Supramolecular nanomedicine constructed from cucurbit[8]uril-based amphiphilic brush copolymer for cancer therapy. ACS Appl Mater Interfaces.

[121] Samanta SK, Moncelet D, Briken V, Isaacs L (2016). Metal-organic polyhedron capped with cucurbit[8]uril delivers doxorubicin to cancer cells. J Am Chem Soc.

[122] Wang Y, Li D, Wang H, Chen Y, Han H, Jin Q, Ji J (2014). pH responsive supramolecular prodrug micelles based on cucurbit[8]uril for intracellular drug delivery. Chem Commun.

[123] Yang Y, Hu H, Chen L, Bai H, Wang S, Xu J-F, Zhang X (2019). Antibacterial supramolecular polymers constructed via self-sorting: promoting antibacterial performance and controllable degradation. Mater Chem Front.

[124] Tian J, Zhou T-Y, Zhang S-C, Aloni S, Altoe MV, Xie S-H, Wang H, Zhang D-W, Zhao X, Liu Y, Li Z-T (2014). Three-dimensional periodic supramolecular organic framework ion sponge in water and microcrystals. Nat Commun.

[125] Huang Z, Yang L, Liu Y, Wang Z, Scherman OA, Zhang X (2014). Supramolecular polymerization promoted and controlled through self-sorting. Angew Chem Int Ed Engl.

[126] Cheng Q, Li S, Ma Y, Yin H, Wang R (2020). pH-responsive supramolecular DOX-dimer based on cucurbit[8]uril for selective drug release. Chinese Chem Lett.

[127] Roy B, Ghosh AK, Srivastava S, D'Silva P, Mukherjee PS (2015). A Pd8 tetrafacial molecular barrel as carrier for water insoluble fluorophore. J Am Chem Soc.

[128] Zhukhovitskiy AV, Zhong M, Keeler EG, Michaelis VK, Sun JEP, Hore MJA, Pochan DJ, Griffin RG, Willard AP, Johnson JA (2016). Highly branched and loop-rich gels via formation of metal-organic cages linked by polymers. Nat Chem.

[129] Wang Q-Q, Gonell S, Leenders SHAM, Dürr M, Ivanović-Burmazović I, Reek JNH (2016). Self-assembled nanospheres with multiple endohedral binding sites pre-organize catalysts and substrates for highly efficient reactions. Nat Chem.

[130] Knopf KM, Murphy BL, MacMillan SN, Baskin JM, Barr MP, Boros E, Wilson JJ (2017). In vitro anticancer activity and in vivo biodistribution of rhenium(I) tricarbonyl aqua complexes. J Am Chem Soc.

[131] Wei P, Yan X, Huang F (2015). Supramolecular polymers constructed by orthogonal self-assembly based on host–guest and metal–ligand interactions. Chem Soc Rev.

[132] Datta S, Misra SK, Saha ML, Lahiri N, Louie J, Pan D, Stang PJ (2018). Orthogonal self-assembly of an organoplatinum(II) metallacycle and cucurbit[8]uril that delivers curcumin to cancer cells. Proc Natl Acad Sci USA.

[133] Nieder C, Grosu AL, Astner S, Molls M (2005). Treatment of unresectable glioblastoma multiforme. Anticancer Res.

[134] Stupp R, Mason WP, van den Bent MJ, Weller M, Fisher B, Taphoorn MJ, Belanger K, Brandes AA, Marosi C, Bogdahn U, Curschmann J, Janzer RC, Ludwin SK, Gorlia T, Allgeier A, Lacombe D, Cairncross JG, Eisenhauer E, Mirimanoff RO (2005). Radiotherapy plus concomitant and adjuvant temozolomide for glioblastoma. N Engl J Med.

[135] Perry J, Chambers A, Spithoff K, Laperriere N (2007). Gliadel wafers in the treatment of malignant glioma: a systematic review. Curr Oncol Rep.

[136] Krex D, Klink B, Hartmann C, von Deimling A, Pietsch T, Simon M, Sabel M, Steinbach JP, Heese O, Reifenberger G, Weller M, Schackert G (2007). Long-term survival with glioblastoma multiforme. Brain.

[137] Chang EL, Akyurek S, Avalos T, Rebueno N, Spicer C, Garcia J, Famiglietti R, Allen PK, Chao KSC, Mahajan A, Woo SY, Maor MH (2007). Evaluation of peritumoral edema in the delineation of radiotherapy clinical target volumes for glioblastoma. Int J Radiat Oncol Biol.

[138] Jackson M, Hassiotou F, Nowak A (2015). Glioblastoma stem-like cells: at the root of tumor recurrence and a therapeutic target. Carcinogenesis.

[139] Rowland MJ, Parkins CC, McAbee JH, Kolb AK, Hein R, Loh XJ, Watts C, Scherman OA (2018). An adherent tissue-inspired hydrogel delivery vehicle utilised in primary human glioma models. Biomaterials.

[140] Østrem RG, Parhamifar L, Pourhassan H, Clergeaud G, Nielsen OL, Kjær A, Hansen AE, Andresen TL (2017). Secretory phospholipase A2 responsive liposomes exhibit a potent anti-neoplastic effect in vitro, but induce unforeseen severe toxicity in vivo. J Control Release.

[141] Chen J, Huang K, Chen Q, Deng C, Zhang J, Zhong Z (2018). Tailor-making fluorescent hyaluronic acid microgels via combining microfluidics and photoclick chemistry for sustained and localized delivery of herceptin in tumors. ACS Appl Mater Interfaces.

[142] Wang C, Chen S, Wang Y, Liu X, Hu F, Sun J, Yuan H (2018). Lipase-triggered water-responsive “pandora’s box” for cancer therapy: toward induced neighboring effect and enhanced drug penetration. Adv Mater.

[143] Grünwald B, Vandooren J, Locatelli E, Fiten P, Opdenakker G, Proost P, Krüger A, Lellouche JP, Israel LL, Shenkman L, Franchini MC (2016). Matrix metalloproteinase-9 (MMP-9) as an activator of nanosystems for targeted drug delivery in pancreatic cancer. J Control Release.

[144] van Rijt SH, Bölükbas DA, Argyo C, Datz S, Lindner M, Eickelberg O, Königshoff M, Bein T, Meiners S (2015). Protease-mediated release of chemotherapeutics from mesoporous silica nanoparticles to ex vivo human and mouse lung tumors. ACS Nano.

[145] Raju GSR, Pavitra E, Varaprasad GL, Bandaru SS, Nagaraju GP, Farran B, Huh YS, Han Y-K (2022). Nanoparticles mediated tumor microenvironment modulation: current advances and applications. J Nanobiotechnol.

[146] Qiao H, Jia J, Shen H, Zhao S, Chen E, Chen W, Di B, Hu C (2019). Capping silica nanoparticles with tryptophan-mediated cucurbit[8]uril complex for targeted intracellular drug delivery triggered by tumor-overexpressed IDO1 enzyme. Adv Healthc Mater.

[147] Zhang Y-M, Zhang N-Y, Xiao K, Yu Q, Liu Y (2018). Photo-controlled reversible microtubule assembly mediated by paclitaxel-modified cyclodextrin. Angew Chem Int Ed.

[148] Rennie ML, Fox GC, Perez J, Crowley PB (2018). Auto-regulated protein assembly on a supramolecular scaffold. Angew Chem Int Ed.

[149] Brouhard GJ, Rice LM (2018). Microtubule dynamics: an interplay of biochemistry and mechanics. Nat Rev Mol Cell Biol.

[150] Bachand GD, Spoerke ED, Stevens MJ (2015). Microtubule-based nanomaterials: exploiting nature's dynamic biopolymers. Biotechnol Bioeng.

[151] Mogaki R, Hashim PK, Okuro K, Aida T (2017). Guanidinium-based, “molecular glues” for modulation of biomolecular functions. Chem Soc Rev.

[152] Zhang Y-M, Liu J-H, Yu Q, Wen X, Liu Y (2019). Targeted polypeptide-microtubule aggregation with cucurbit[8]uril for enhanced cell apoptosis. Angew Chem Int Ed Engl.

[153] Dolmans DE, Fukumura D, Jain RK (2003). Photodynamic therapy for cancer. Nat Rev Cancer.

[154] Roy I, Shetty D, Hota R, Baek K, Kim J, Kim C, Kappert S, Kim KA (2015). Multifunctional subphthalocyanine nanosphere for targeting, labeling, and killing of antibiotic-resistant bacteria. Angew Chem Int Ed.

[155] Castano AP, Mroz P, Hamblin MR (2006). Photodynamic therapy and anti-tumour immunity. Nat Rev Cancer.

[156] He P, Yang G, Zhu D, Kong H, Corrales-Ureña YR, Ciacchi LC, Wei G (2022). Biomolecule-mimetic nanomaterials for photothermal and photodynamic therapy of cancers: bridging nanobiotechnology and biomedicine. J Nanobiotechnol.

[157] Guo S, Gu D, Yang Y, Tian J, Chen X (2023). Near-infrared photodynamic and photothermal co-therapy based on organic small molecular dyes. J Nanobiotechnol.

[158] Meng Z, Xue H, Wang T, Chen B, Dong X, Yang L, Dai J, Lou X, Xia F (2022). Aggregation-induced emission photosensitizer-based photodynamic therapy in cancer: from chemical to clinical. J Nanobiotechnol.

[159] He J, Wang Y, Missinato MA, Onuoha E, Perkins LA, Watkins SC, St Croix CM, Tsang M, Bruchez MP (2016). A genetically targetable near-infrared photosensitizer. Nat Methods.

[160] Durantini AM, Greene LE, Lincoln R, Martínez SR, Cosa G (2016). Reactive oxygen species mediated activation of a dormant singlet oxygen photosensitizer: from autocatalytic singlet oxygen amplification to chemicontrolled photodynamic therapy. J Am Chem Soc.

[161] Yuan Y, Zhang C-J, Gao M, Zhang R, Tang B, Liu B (2015). Specific light-up bioprobe with aggregation-induced emission and activatable photoactivity for the targeted and image-guided photodynamic ablation of cancer cells. Angew Chem Int Ed.

[162] Yuan Y, Xu S, Zhang CJ, Zhang R, Liu B (2016). Dual-targeted activatable photosensitizers with aggregation-induced emission (AIE) characteristics for image-guided photodynamic cancer cell ablation. J Mater Chem B.

[163] Horiuchi H, Kuribara R, Hirabara A, Okutsu T (2016). pH-response optimization of amino-substituted tetraphenylporphyrin derivatives as pH-activatable photosensitizers. J Phys Chem A.

[164] Wang X-Q, Lei Q, Zhu J-Y, Wang W-J, Cheng Q, Gao F, Sun Y-X, Zhang X-Z (2016). Cucurbit[8]uril regulated activatable supramolecular photosensitizer for targeted cancer imaging and photodynamic therapy. ACS Appl Mater Interfaces.

[165] Li X, Kim J, Yoon J, Chen X (2017). Cancer-associated, stimuli-driven, turn on theranostics for multimodality imaging and therapy. Adv Mater.

[166] Li X, Kim C-Y, Lee S, Lee D, Chung H-M, Kim G, Heo S-H, Kim C, Hong K-S, Yoon J (2017). Nanostructured phthalocyanine assemblies with protein-driven switchable photoactivities for biophotonic imaging and therapy. J Am Chem Soc.

[167] Lü B, Chen Y, Li P, Wang B, Müllen K, Yin M (2019). Stable radical anions generated from a porous perylenediimide metal-organic framework for boosting near-infrared photothermal conversion. Nat Commun.

[168] Lin H, Gao S, Dai C, Chen Y, Shi J (2017). A two-dimensional biodegradable niobium carbide (MXene) for photothermal tumor eradication in NIR-I and NIR-II biowindows. J Am Chem Soc.

[169] Jiang Y, Li J, Zhen X, Xie C, Pu K (2018). Dual-peak absorbing semiconducting copolymer nanoparticles for first and second near-infrared window photothermal therapy: a comparative study. Adv Mater.

[170] Xu G, Yan Q, Lv X, Zhu Y, Xin K, Shi B, Wang R, Chen J, Gao W, Shi P, Fan C, Zhao C, Tian H (2018). Imaging of colorectal cancers using activatable nanoprobes with second near-infrared window emission. Angew Chem Int Ed.

[171] Tang B, Li W-L, Chang Y, Yuan B, Wu Y, Zhang M-T, Xu J-F, Li J, Zhang X (2019). A supramolecular radical dimer: high-efficiency NIR-II photothermal conversion and therapy. Angew Chem Int Ed Engl.

[172] Wang W, Wang X, Cao J, Liu J, Qi B, Zhou X, Zhang S, Gabel D, Nau WM, Assaf KI, Zhang H (2018). The chaotropic effect as an orthogonal assembly motif for multi-responsive dodecaborate-cucurbituril supramolecular networks. Chem Commun.

[173] Qi B, Li X, Sun L, Chen B, Chen H, Wu C, Zhang H, Zhou X (2018). In situ synthesis of ultrafine metal clusters triggered by dodecaborate supramolecular organic frameworks. Nanoscale.

[174] Qi B, Du L, Yao F, Xu S, Deng X, Zheng M, He S, Zhang H, Zhou X (2019). Shape-controlled dodecaborate supramolecular organic-framework-supported ultrafine trimetallic PtCoNi for catalytic hydrolysis of ammonia borane. ACS Appl Mater Interfaces.

[175] Wang W, Qi B, Yu X, Li W-Z, Yang Z, Zhang H, Liu S, Liu Y, Wang X-Q (2020). Modular design of supramolecular organic frameworks for image-guided photodynamic therapy. Adv Funct Mater.

[176] Kessel D (2019). Photodynamic therapy: a brief history. J Clin Med.

[177] Jiang Z, He L, Yu X, Yang Z, Wu W, Wang X, Mao R, Cui D, Chen X, Li W (2021). Antiangiogenesis combined with inhibition of the hypoxia pathway facilitates lowdose. X-ray-induced photodynamic therapy ACS Nano.

[178] Gao J, Li J, Geng WC, Chen FY, Duan X, Zheng Z, Ding D, Guo DS (2018). Biomarker displacement activation: a general host–guest strategy for targeted phototheranostics in vivo. J Am Chem Soc.

[179] Allison RR, Sibata CH (2010). Oncologic photodynamic therapy photosensitizers: a clinical review. Photodiagnosis Photodyn Ther.

[180] Sadzuka Y, Tokutomi K, Iwasaki F, Sugiyama I, Hirano T, Konno H, Oku N, Sonobe T (2006). The phototoxicity of photofrin was enhanced by PEGylated liposome in vitro. Cancer Lett.

[181] Liu Y, Liu C-Z, Wang Z-K, Zhou W, Wang H, Zhang Y-C, Zhang D-W, Ma D, Li Z-T (2022). Supramolecular organic frameworks improve the safety of clinically used porphyrin photodynamic agents and maintain their antitumor efficacy. Biomaterials.

[182] Tian F, Jiao D, Biedermann F, Scherman OA (2012). Orthogonal switching of a single supramolecular complex. Nat Commun.

[183] del Barrio J, Horton PN, Lairez D, Lloyd GO, Toprakcioglu C, Scherman OA (2013). Photocontrol over cucurbit[8]uril complexes: stoichiometry and supramolecular polymers. J Am Chem Soc.

[184] Hubbell JA (2003). Enhancing drug function. Science.

[185] Hamley IW, Castelletto V (2007). Biological soft materials. Angew Chem Int Ed.

[186] Schuster GB (2000). Long-range charge transfer in DNA: transient structural distortions control the distance dependence. Acc Chem Res.

[187] Cheng H-B, Zhang Y-M, Xu C, Liu Y (2014). Photoresponsive supramolecular complexes as efficient DNA regulator. Sci Rep.

[188] Ren Y, Li RQ, Cai YR, Xia T, Yang M, Xu F (2016). Effective codelivery of incRNA and pDNA by pullulan-based nanovectors for promising therapy of hepatocellular carcinoma. Adv Funct Mater.

[189] Au SL-K, Wong CC-L, Lee JM-F, Fan DN-Y, Tsang FH, Ng IO-L, Wong C-M (2012). Enhancer of zeste homolog 2 epigenetically silences multiple tumor suppressor microRNAs to promote liver cancer metastasis. Hepatology.

[190] Xu L, Beckebaum S, Iacob S, Wu G, Kaiser GM, Radtke A, Liu C, Kabar I, Schmidt HH, Zhang X, Lu M, Cicinnati VR (2014). MicroRNA-101 inhibits human hepatocellular carcinoma progression through EZH2 downregulation and increased cytostatic drug sensitivity. J Hepatol.

[191] Chien Y, Hsiao Y-J, Chou S-J, Lin T-Y, Yarmishyn AA, Lai W-Y, Lee M-S, Lin Y-Y, Lin T-W, Hwang D-K, Lin T-C, Chiou S-H, Chen S-J, Yang Y-P (2022). Nanoparticles-mediated CRISPR-Cas9 gene therapy in inherited retinal diseases: applications, challenges, and emerging opportunities. J Nanobiotechnol.

[192] Zhou D, Gao Y, Aied A, Cutlar L, Igoucheva O, Newland B, Alexeeve V, Greiser U, Uitto J, Wang W (2016). Highly branched poly(β-amino ester)s for skin gene therapy. J Controlled Release.

[193] Zhou D, Cutlar L, Gao Y, Wang W, O'Keeffe-Ahern J, McMahon S, Duarte B, Larcher F, Rodriguez BJ, Greiser U, Wang W (2016). The transition from linear to highly branched poly(β-amino ester)s: branching matters for gene delivery. Sci Adv.

[194] Song H-Q, Pan W, Li R-Q, Yu B, Liu W, Yang M, Xu F-J (2018). Rodlike supramolecular nanoassemblies of degradable poly(aspartic acid) derivatives and hydroxyl-rich polycations for effective delivery of versatile tumor-suppressive ncRNAs. Small.

[195] Agrawal N, Dasaradhi PVN, Mohmmed A, Malhotra P, Bhatnagar RK, Mukherjee SK (2003). RNA interference: biology, mechanism, and applications. Microbiol Mol Biol Rev.

[196] Davis ME, Zuckerman JE, Choi CHJ, Seligson D, Tolcher A, Alabi CA, Yen Y, Heidel JD, Ribas A (2010). Evidence of RNAi in humans from systemically administered siRNA via targeted nanoparticles. Nature.

[197] Elbashir SM, Harborth J, Lendeckel W, Yalcin A, Weber K, Tuschl T (2001). Duplexes of 21-nucleotide RNAs mediate RNA interference in cultured mammalian cells. Nature.

[198] Castanotto D, Rossi JJ (2009). The promises and pitfalls of RNA-interference-based therapeutics. Nature.

[199] Li L, He W, You W, Yan J, Liu W (2022). Turing miRNA into infinite coordination supermolecule: a general and enabling nanoengineering strategy for resurrecting nuclear acid therapeutics. J Nanobiotechnol.

[200] Li F, Wang M, Guan S, Huang Z, Liu S, Li X, Jiang X, Luo Q, Xu J, Liu J (2019). Cucurbit[8]uril-based supramolecular polymer nanocapsules as an effective siRNA delivery platform for gene therapy. Polym Chem.

[201] Hoffmann-Röder A, Kaiser A, Wagner S, Gaidzik N, Kowalczyk D, Westerlind U, Gerlitzki B, Schmitt E, Kunz H (2010). Synthetic antitumor vaccines from tetanus toxoid conjugates of MUC1 glycopeptides with the Thomsen-Friedenreich antigen and a fluorine-substituted analogue. Angew Chem Int Ed Engl.

[202] Li X, Fujio M, Imamura M, Wu D, Vasan S, Wong C-H, Ho DD, Tsuji M (2010). Design of a potent CD1d-binding NKT cell ligand as a vaccine adjuvant. Proc Natl Acad Sci USA.

[203] Alexopoulou L, Thomas V, Schnare M, Lobet Y, Anguita J, Schoen RT, Medzhitov R, Fikrig E, Flavell RA (2002). Hyporesponsiveness to vaccination with borrelia burgdorferi OspA in humans and in TLR1- and TLR2-deficient mice. Nat Med.

[204] Wright TH, Brooks AE, Didsbury AJ, Williams GM, Harris PW, Dunbar P, Brimble MA (2013). Direct peptide lipidation through thiol-ene coupling enables rapid synthesis and evaluation of self-adjuvanting vaccine candidates. Angew Chem Int Ed Engl.

[205] Liao Z, Huang J, Lo P-C, Lovell JF, Jin H, Yang K (2022). Self-adjuvanting cancer nanovaccines. J Nanobiotechnol.

[206] Gao Y, Sun Z-Y, Huang Z-H, Chen P-G, Chen Y-X, Zhao Y-F, Li Y-M (2014). Covalent bond or noncovalent bond: a supramolecular strategy for the construction of chemically synthesized vaccines. Chem Eur J.

[207] Kong HH, Segre JA (2020). Cultivating fungal research. Science.

[208] McLellan CA, Vincent BM, Solis NV, Lancaster AK, Sullivan LB, Hartland CL, Youngsaye W, Filler SG, Whitesell L, Lindquist S (2018). Inhibiting mitochondrial phosphate transport as an unexploited antifungal strategy. Nat Chem Biol.

[209] Fukushima K, Liu S, Wu H, Engler AC, Coady DJ, Maune H, Pitera J, Nelson A, Wiradharma N, Venkataraman S, Huang Y, Fan W, Ying JY, Yang YY, Hedrick JL (2013). Supramolecular high-aspect ratio assemblies with strong antifungal activity. Nat Commun.

[210] Li Q, Li J, Yu W, Wang Z, Li J, Feng X, Wang J, Shan A (2021). De novo design of a pH-triggered self-assembled β-hairpin nanopeptide with the dual biological functions for antibacterial and entrapment. J Nanobiotechnol.

[211] Fisher MC, Hawkins NJ, Sanglard D, Gurr SJ (2018). Worldwide emergence of resistance to antifungal drugs challenges human health and food security. Science.

[212] Li Q, Li Y, Min T, Gong J, Du L, Phillips DL, Liu J, Lam JWY, Sung HHY, Williams ID, Kwok RTK, Ho CL, Li K, Wang J, Tang B (2020). Time-dependent photodynamic therapy for multiple targets: a highly efficient AIE-active photosensitizer for selective bacterial elimination and cancer cell ablation. Angew Chem Int Ed.

[213] Hu F, Xu S, Liu B (2018). Photosensitizers with aggregation-induced emission: materials and biomedical applications. Adv Mater.

[214] Zhu W, Li Y, Guo S, Guo W-J, Peng T, Li H, Liu B, Peng H-Q, Tang B (2022). Stereoisomeric engineering of aggregation-induced emission photosensitizers towards fungal killing. Nat Commun.

[215] Rillahan CD, Paulson JC (2011). Glycan microarrays for decoding the glycome. Annu Rev Biochem.

[216] Jonkheijm P, Weinrich D, Schröder H, Niemeyer CM, Waldmann H (2008). Chemical strategies for generating protein biochips. Angew Chem Int Ed.

[217] Voskuhl J, Brinkmann J, Jonkheijm P (2014). Advances in contact printing technologies of carbohydrate, peptide and protein arrays. Curr Opin Chem Biol.

[218] Sankaran S, van Weerd J, Voskuhl J, Karperien M, Jonkheijm P (2015). Photoresponsive cucurbit[8]uril-mediated adhesion of bacteria on supported lipid bilayers. Small.

[219] Mhammedi MAEI, Bakasse M, Chtaini A (2007). Electrochemical studies and square wave voltammetry of paraquat at natural phosphate modified carbon paste electrode. J Hazard Mater.

[220] Diaz S, Martín-González A, Cubas L, Ortega R, Amaro F, Rodríguez-Martín D, Gutiérrez J-C (2016). High resistance of tetrahymena thermophila to paraquat: mitochondrial alterations, oxidative stress and antioxidant genes expression. Chemosphere.

[221] Dinis-Oliveira RJ, Duarte JA, Sánchez-Navarro A, Remião F, Bastos ML, Carvalho F (2008). Paraquat poisonings: mechanisms of lung toxicity, clinical features, and treatment. Crit Rev Toxicol.

[222] Kniss AR (2017). Long-term trends in the intensity and relative toxicity of herbicide use. Nat Commun.

[223] Gao C, Huang Q, Lan Q, Feng Y, Tang F, Hoi MPM, Zhang J, Lee SMY, Wang R (2018). A user-friendly herbicide derived from photo-responsive supramolecular vesicles. Nat Commun.

[224] Gao C, Kwong CHT, Sun C, Li Z, Lu S, Yang Y-W, Lee SMY, Wang R (2020). Selective decoating-induced activation of supramolecularly coated toxic nanoparticles for multiple applications. ACS Appl Mater Interfaces.

[225] Chen DS, Mellman I (2017). Elements of cancer immunity and the cancer-immune set point. Nature.

[226] Prendergast GC, Malachowski WP, DuHadaway JB, Muller A (2017). Discovery of IDO1 inhibitors: from bench to bedside. Cancer Res.

[227] Godin-Ethier J, Hanafi L-A, Piccirillo CA, Lapointe R (2011). Indoleamine 2,3-dioxygenase expression in human cancers: clinical and immunologic perspectives. Clin Cancer Res.

[228] Gu K, Xu Y, Li H, Guo Z, Zhu S, Zhu S, Shi P, James TD, Tian H, Zhu W-H (2016). Real-time tracking and in vivo visualization of β-galactosidase activity in colorectal tumor with a ratiometric near-infrared fluorescent probe. J Am Chem Soc.

[229] Tian Z, Ding L, Li K, Song Y, Dou T, Hou J, Tian X, Feng L, Ge G, Cui J (2019). Rational design of a long-wavelength fluorescent probe for highly selective sensing of carboxylesterase 1 in living systems. Anal Chem.

[230] Takikawa O, Kuroiwa T, Yamazaki F, Kido R (1988). Mechanism of interferon-gamma action. Characterization of indoleamine 2,3-dioxygenase in cultured human cells induced by interferon-gamma and evaluation of the enzyme-mediated tryptophan degradation in its anticellular activity. J Biol Chem.

[231] Tomek P, Palmer BD, Flanagan JU, Sun C, Raven EL, Ching L-M (2017). Discovery and evaluation of inhibitors to the immunosuppressive enzyme indoleamine 2,3-dioxygenase 1 (IDO1): probing the active site-inhibitor interactions. Eur J Med Chem.

[232] Seegers N, van Doornmalen AM, Uitdehaag JCM, de Man J, Buijsman RC, Zaman GJR (2014). High-throughput fluorescence-based screening assays for tryptophan-catabolizing enzymes. J Biomol Screen.

[233] Jia J, Wen H, Zhao S, Wang L, Qiao H, Shen H, Yu Z, Di B, Xu L, Hu C (2019). Displacement induced off-on fluorescent biosensor targeting IDO1 activity in live cells. Anal Chem.

[234] Weißenstein A, Saha-Möller CR, Würthner F (2018). Optical sensing of aromatic amino acids and dipeptides by a crown-etherfunctionalized perylene bisimide fluorophore. Chem Eur J.

[235] Huang Q, Jiang L, Liang W, Gui J, Xu D, Wu W, Nakai Y, Nishijima M, Fukuhara G, Mori T, Inoue Y, Yang C (2016). Inherently chiral azonia[6]helicene-modified β-cyclodextrin: synthesis, characterization, and chirality sensing of underivatized amino acids in water. J Org Chem.

[236] Ghale G, Nau WM (2014). Dynamically analyte-responsive macrocyclic host–fluorophore systems. Acc Chem Res.

[237] Yu X, Liang W, Huang Q, Wu W, Chruma JJ, Yang C (2019). Room-temperature phosphorescent γ-cyclodextrincucurbit[6]uril-cowheeled [4]rotaxanes for specific sensing of tryptophan. Chem Commun.

[238] Krämer J, Grimm LM, Zhong C, Hirtz M, Biedermann F (2023). A supramolecular cucurbit[8]uril-based rotaxane chemosensor for the optical tryptophan detection in human serum and urine. Nat Commun.

[239] Bai Q, Xia Y, Kang Y, Jiang Y, Hu J, Ma P, Tao Z, Xiao X (2023). A new supramolecular fluorescent probe based on cucurbit[8]uril for visual determination of norfloxacin: multi-color, quantitative detection, and practical applications. Chem Eng J.

[240] Zhang W, Luo Y, Liu C, Yang M-X, Gou J-X, Huang Y, Ni X-L, Tao Z, Xiao X (2022). Supramolecular room temperature phosphorescent materials based on cucurbit[8]uril for dual detection of dodine. ACS Appl Mater Interfaces.

